# Supplementary material for: Gut microbiome of the largest living rodent harbors unprecedented enzymatic systems to degrade plant polysaccharides
Source: Nat Commun. 2022 Feb 2;13:629. doi: 10.1038/s41467-022-28310-y (PMC8810776; doi:10.1038/s41467-022-28310-y)
Supplement: Supplementary file 1 — Supplementary information [file 41467_2022_28310_MOESM1_ESM.pdf]

SUPPLEMENTARY INFORMATION

Gut microbiome of the largest living rodent harbors unprecedented enzymatic systems to degrade plant polysaccharides

Lucelia Cabral[1#], Gabriela F. Persinoti[1#*], Douglas A. A. Paixão[1#], Marcele P. Martins[1,2], Mariana A. B. Morais[1], Mariana Chinaglia[1,2], Mariane N. Domingues[1], Mauricio L. Sforca[3], Renan A. S. Pirolla[1], Wesley C. Generoso[1], Clelton A. dos Santos[1], Lucas F. Maciel[1], Nicolas Terrapon[4,5], Vincent Lombard[4,5], Bernard Henrissat[6,7], Mario T. Murakami[1*]

[1]Brazilian Biorenewables National Laboratory, Brazilian Center for Research in Energy and Materials, Campinas, São Paulo, Brazil.

[2]Graduate Program in Functional and Molecular Biology, Institute of Biology, University of Campinas, Campinas, SP, Brazil

[3]Brazilian Biosciences National Laboratory, Brazilian Center for Research in Energy and Materials, Campinas, São Paulo, Brazil.

[4]The Institut National de la Recherche Agronomique, USC 1408 AFMB, F-13288 Marseille, France.

[5]Architecture et Fonction des Macromolécules Biologiques, CNRS, Aix-Marseille Université, Marseille, France.

[6]Department of Biotechnology and Biomedicine (DTU Bioengineering), Technical University of Denmark, DK-2800 Kgs. Lyngby, Denmark

[7]Department of Biological Sciences, King Abdulaziz University, Jeddah, Saudi Arabia.

[#]These authors contributed equally: Cabral L, Persinoti GF, Paixão DAA.

*Correspondence should be addressed to M.T.M. (mario.murakami@lnbr.cnpem.br) and G.F.P. (gabriela.persinoti@lnbr.cnpem.br)

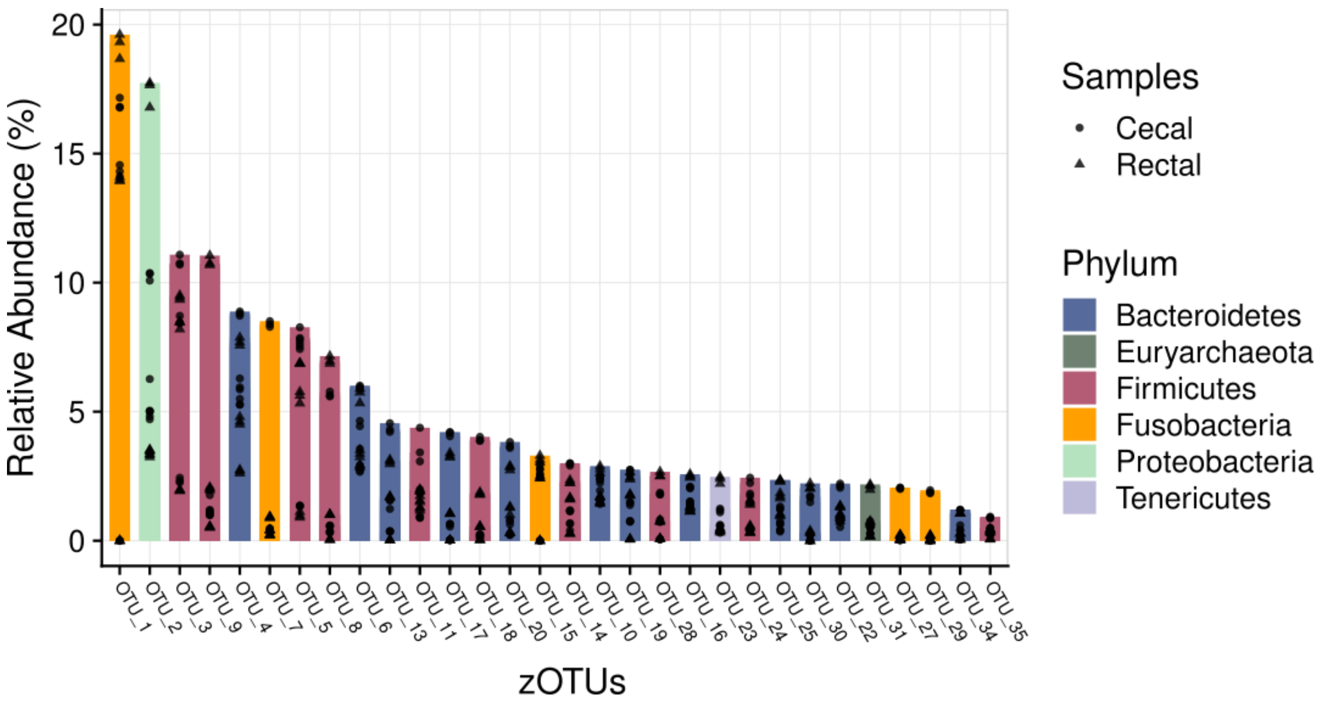

Supplementary Fig. 1. Top-most abundant zOTUs (Exact Operational Taxonomic Units) identified by 16S rRNA gene target sequencing in capybara cecal and rectal samples. The data points were obtained from three independent experiments with three technical replicates, using fresh samples collected from the cecum and recto of three wild animals by abdominal surgical procedure. Source data are provided as a Source Data file.

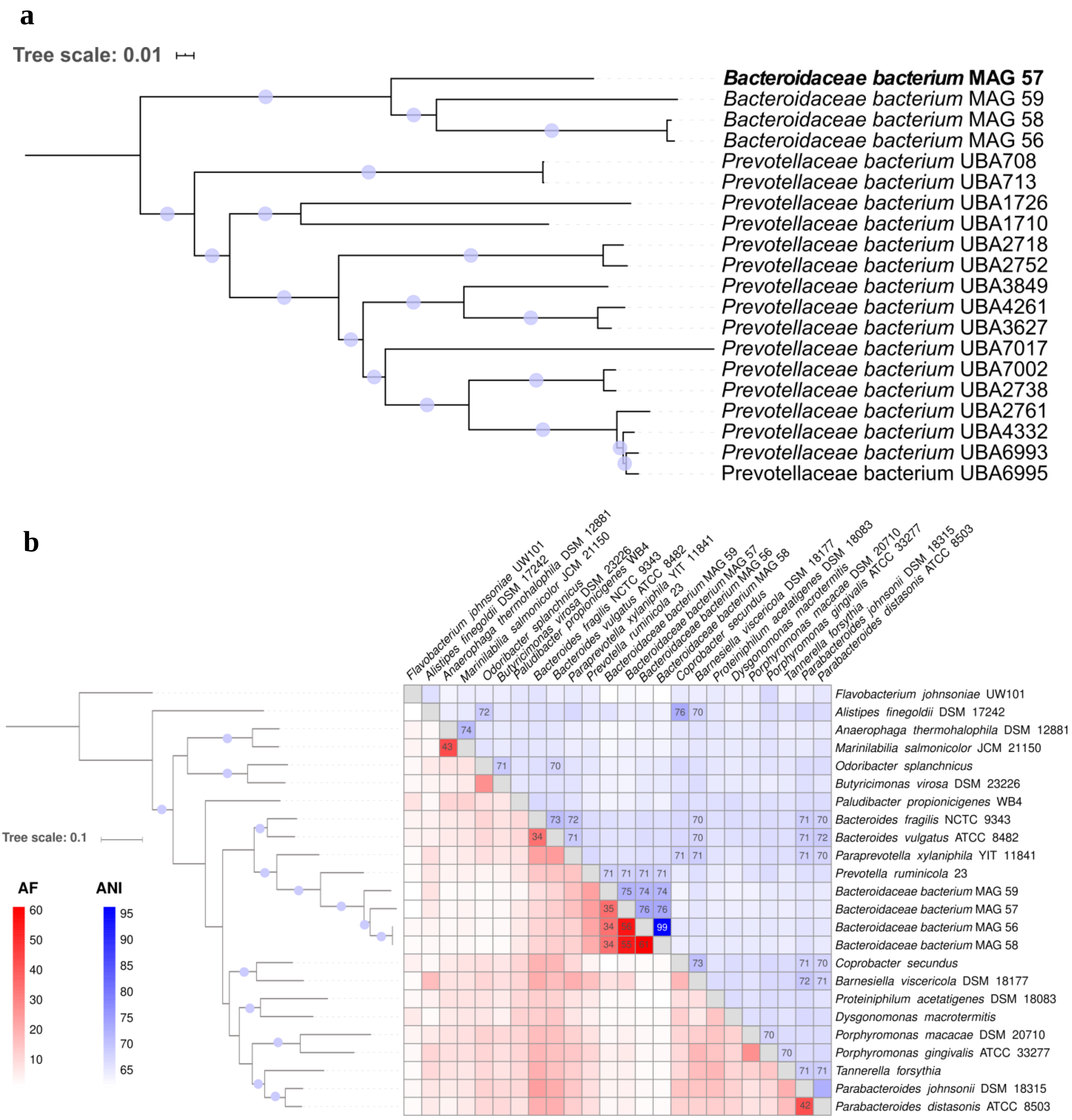

Supplementary Fig. 2. Genome-wide comparisons of *Bacteroidaceae bacterium* MAG57 genome. **a**, Phylogenetic relationship of *Bacteroidaceae bacterium* MAG57 genome and Bacteroidaceae uncultured genomes recovered from the UBA project[1]. Nodes with support values > 80 are indicated by light blue circles. **b**, Average nucleotide identity (blue) and alignment fraction (red) values calculated for reference Bacteroidetes genomes and *Bacteroidaceae bacterium* sp. MAG57 coupled with a multi-locus phylogenetic analysis based on concatenated 92 single copy core genes according to UBCG method[2], using Flavobetacteriaceae as outgroup. Nodes support values > 80 are shown.

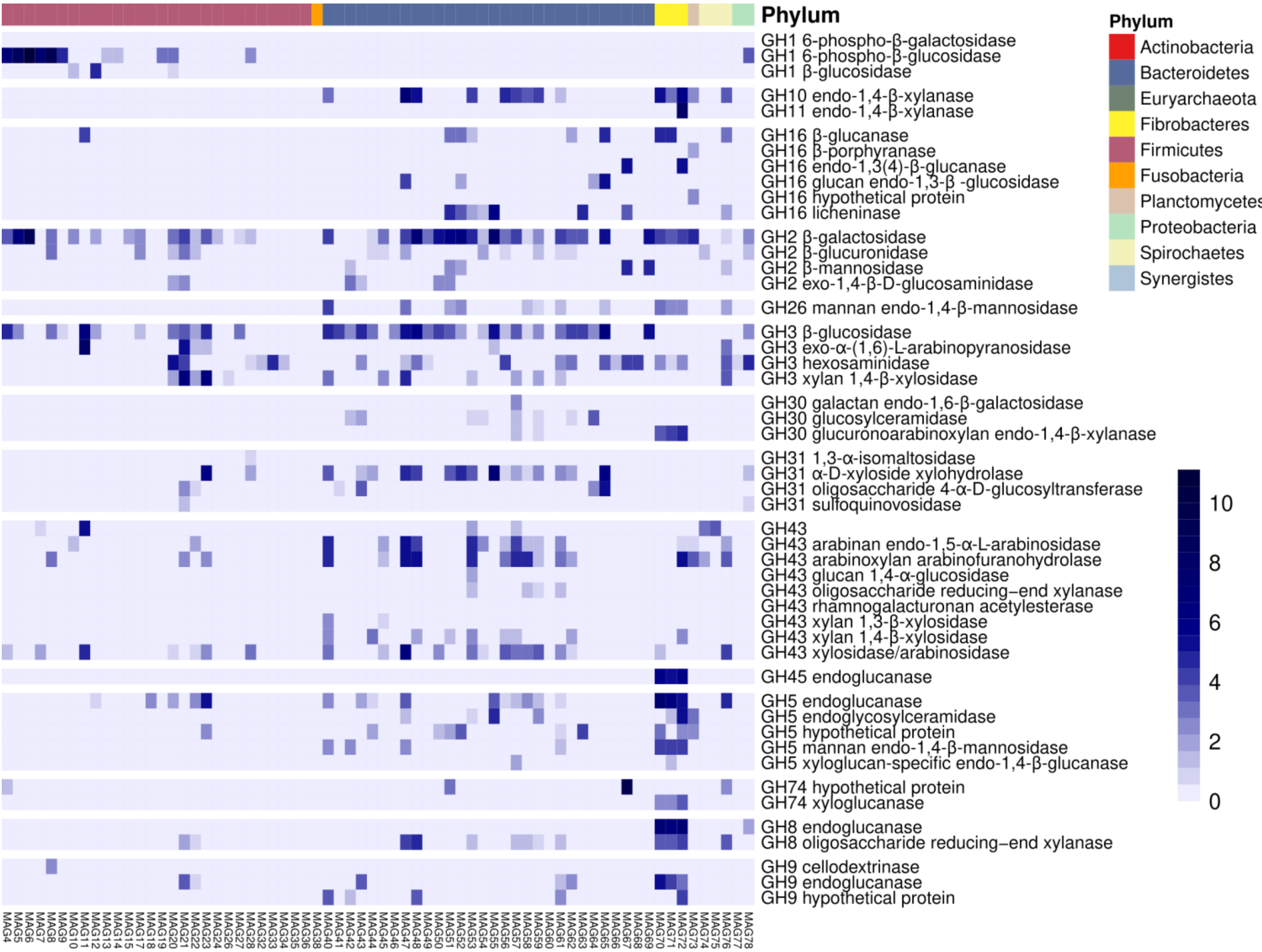

Supplementary Fig. 3. Abundance of expressed Carbohydrate-Active Enzymes (CAZymes) in the recovered Metagenome-Assembled Genomes (MAGs). For each MAG, the expression of protein-coding genes associated with the same activity within a CAZy family were added up and log-normalized. Individual genes and their corresponding expression values (Transcripts Per Million - TPM) are shown in Supplementary Data 4. Source data are provided as a Source Data file.

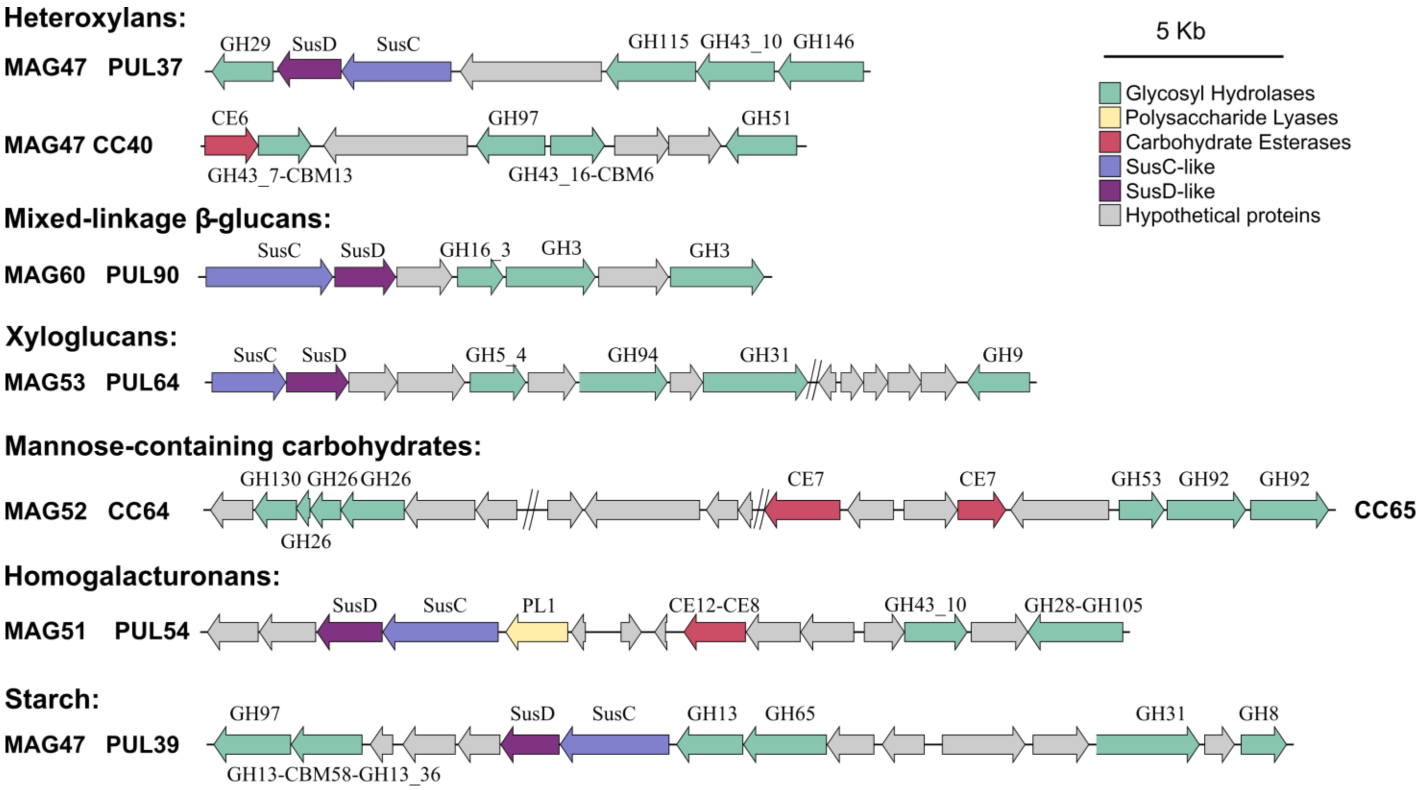

Supplementary Fig. 4. Main carbohydrate degradation systems: Polysaccharide Utilization Loci (PULs) and Clusters of CAZymes (CCs) identified in the capybara metagenome-assembled genomes (MAGs). Colored and gray arrows indicate identified and non-identified CAZyme sequences, respectively.

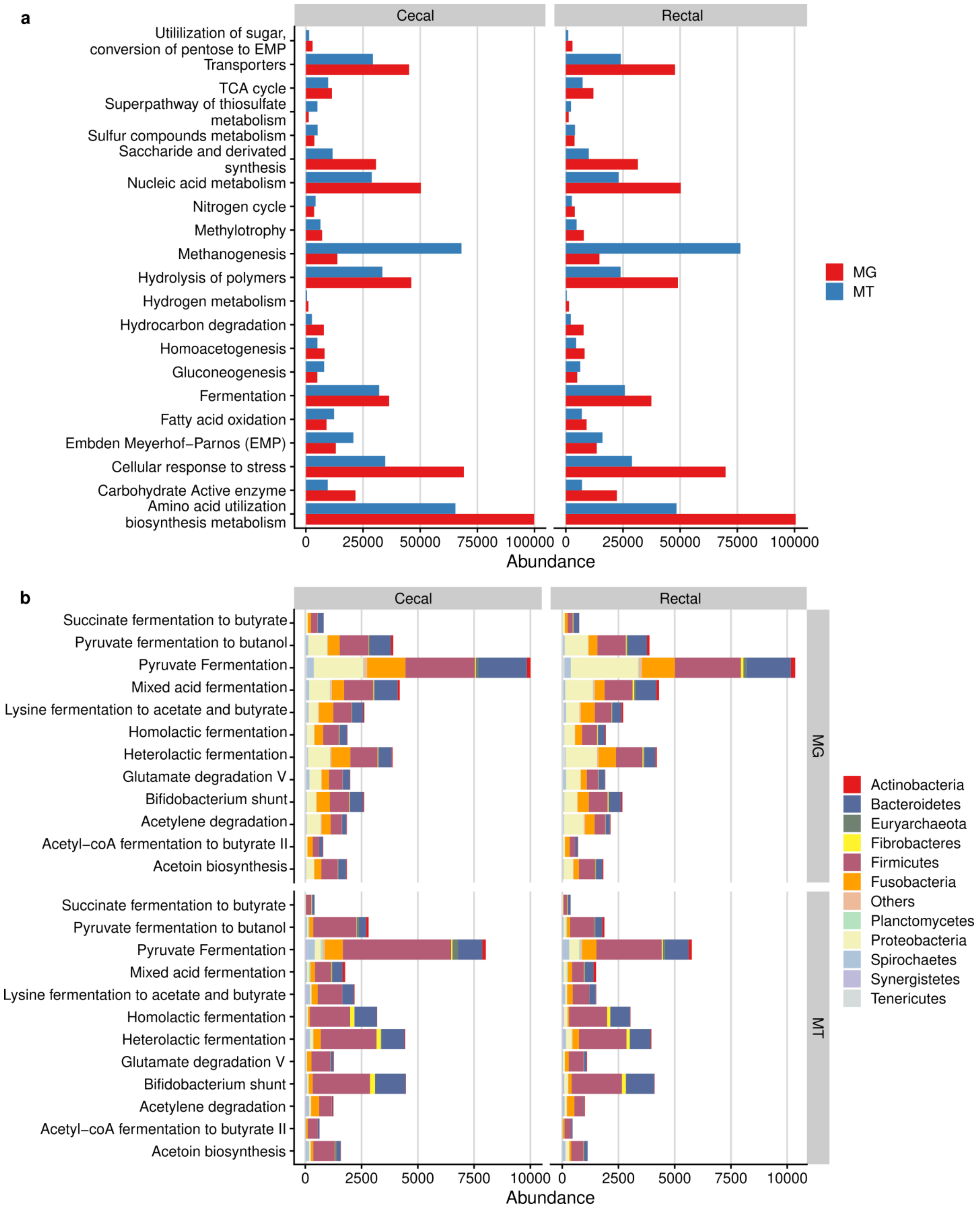

Supplementary Fig. 5. Functional annotation of Capybara metagenome co-assembly predicted genes according to FOAM database. a, Cumulative abundance of main categories. **b**, Cumulative abundance of fermentation-related categories. MG: Metagenome; MT: Metatranscriptome; Abundance is expressed as the cumulative TPM (Transcripts per Million). Source data are provided as a Source Data file.

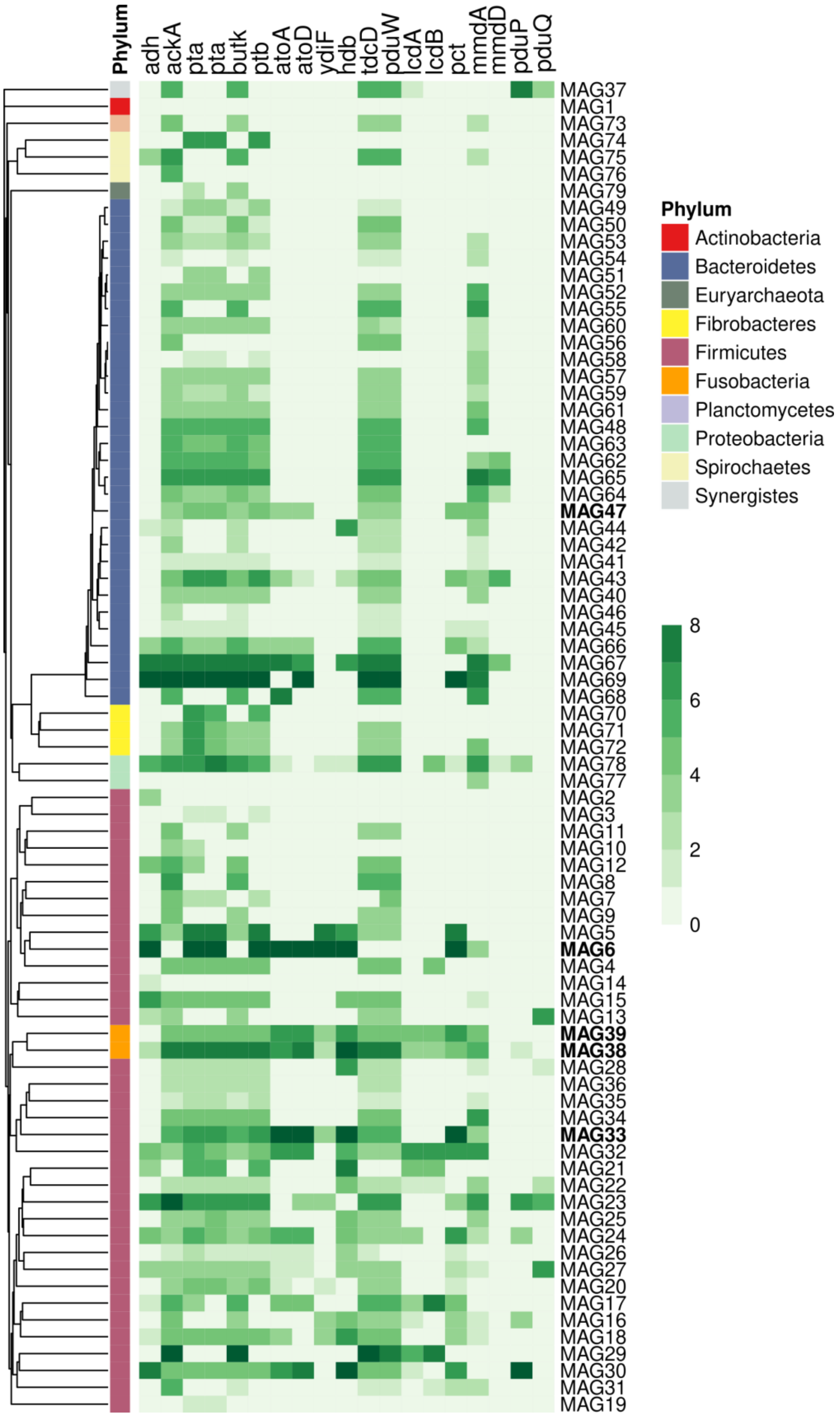

Supplementary Fig. 6. Expression of genes related to dietary components fermentation into Short Chain Fatty Acids (SCFAs). For each Metagenome-assembled genome (MAG), the expression of protein-coding genes associated with the same KEGG Orthologous (KO) annotation were added up and log-normalized. Individual genes and their corresponding expression values (TPM – Transcripts Per Million) are shown in the Supplementary Data 7. Genes encoding products - *adh*: alcohol dehydrogenase; *ackA*: acetate kinase; *pta*: phosphate acetyltransferase; *butK*: butyrate kinase; *ptb*: phosphate butyryltransferase; *atoA*: Butyryl-CoA:acetate CoA-transferase; *atoD*: Butyryl-CoA:acetate CoA-transferase; *ydiF*: acetate CoA/acetoacetate CoA-transferase; *hbd*: butyryl coA dehydrogenase; *tdcD*: propionate kinase; *pduW*: propionate kinase; *lcdA*: lactoyl-CoA dehydratase subunit alpha; *lcdB*: lactoyl-CoA dehydratase subunit beta; *pct*: propionate CoA-transferase; *mmdA*: methylmalonyl coA decarboxylase; *mmdD*: methylmalonyl coA decarboxylase; *pduP:* propionaldehyde dehydrogenase; and *pduQ*: propanol dehydrogenase. Source data are provided as a Source Data file.

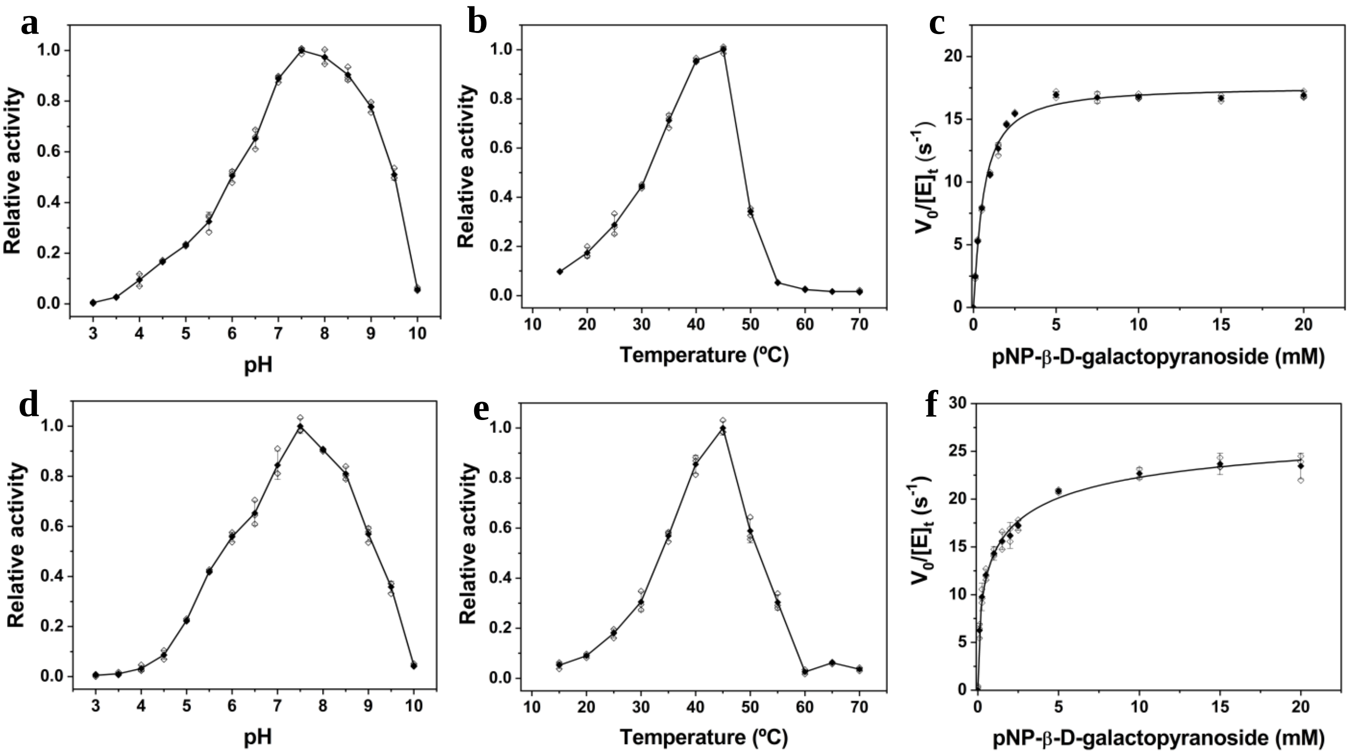

Supplementary Fig. 7. Biochemical and kinetic characterization of the founding members of the GH173 Family. Effects of pH and temperature on relative activity and kinetic curves. **a-c,** CapGH173 (PBMDCECB_44807). **d-f**, BXY_26070 **(**CBK67650.1). Both enzymes were assayed against pNP-β-D-Galactopyranoside at pH 7.5 and 45 °C. Kinetic parameters are presented in Table 1. Results are expressed as mean ± SD from three independent experiments (n=3). Data points are shown as empty symbols. Source data are provided as a Source Data file.

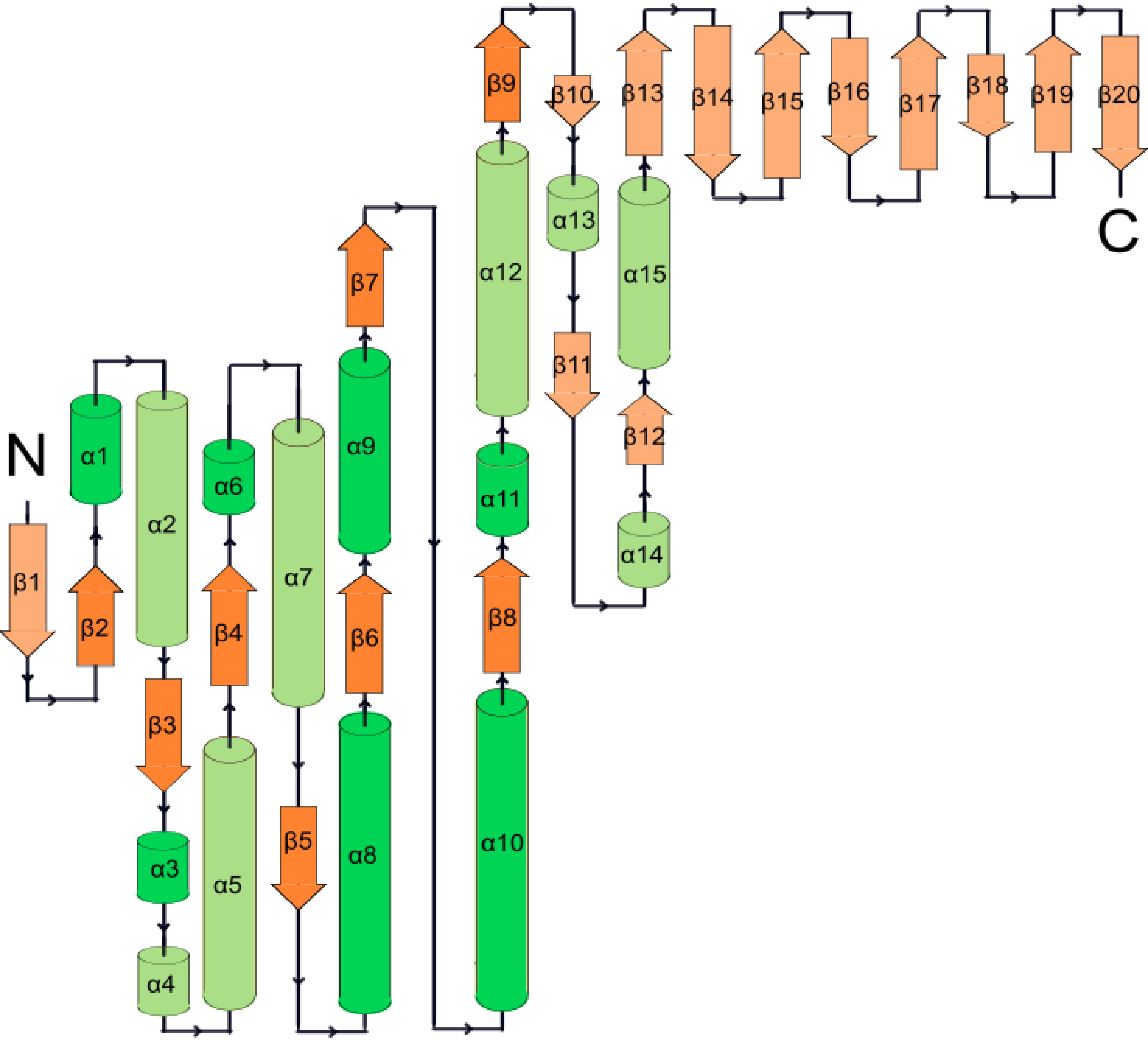

Supplementary Fig. 8. Prediction of the structural topology of the GH173 family according to RoseTTAFold[3] structural model. The predictor generated a model with 0.74 of confidence, and the protein topology was obtained using PDBsum[4]. The secondary structural elements forming the (α/β)-barrel are represented by dark colors.

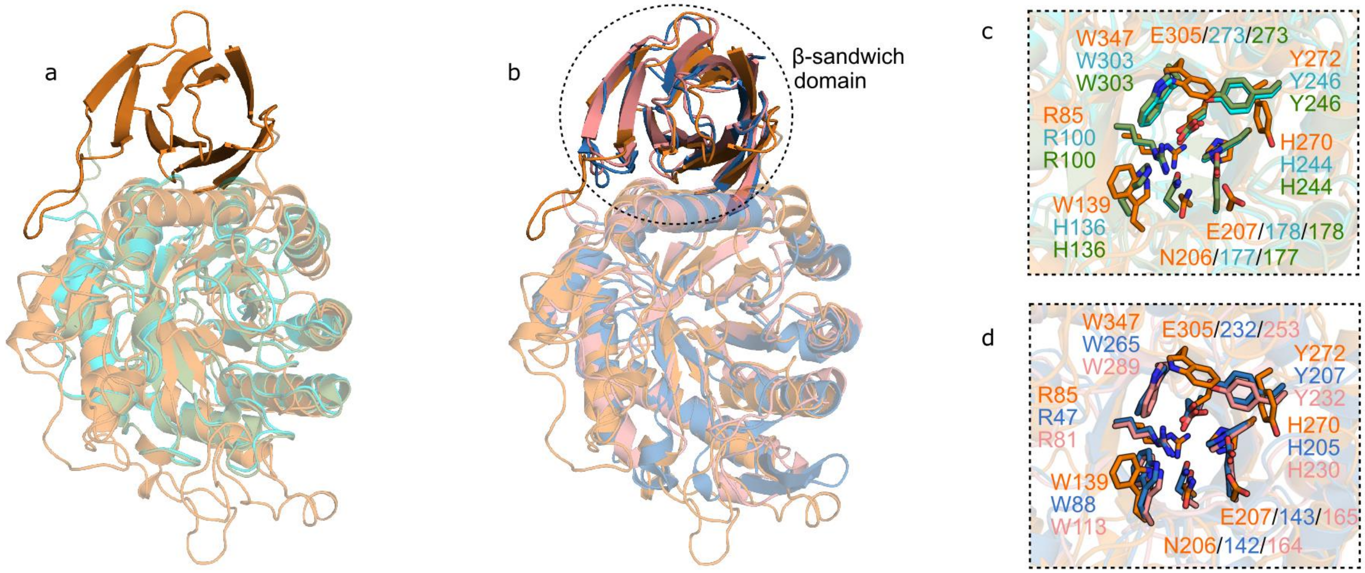

Supplementary Fig. 9. Structural comparison between CapGH173 model and (a) GH5 and (b) GH30 members. GH5 members are the endo-β-1,4-mannanases from *Streptomyces thermolilacinus* NBRC14274 (StMan, subfamily GH5_8, PDB code 3WSU, rmsd 3.27 Å, in cyan[5]) and from *Streptomyces* sp. SirexAA-E (SACTE_2347, subfamily GH5_8, PDB code 4FK9, 4.41 Å rmsd, in green[6]). GH30 members are the endo-β-1,4-xylanase from *Ruminiclostridium papyrosolvens* C71 (CpXyn30A, subfamily GH30_8, PDB code 4FMV, rmsd 2.07 Å, in blue[7]) and the glucuronoarabinoxylan-specific endo-β-1,4-xylanase from *Erwinia chrysanthemi* (XynA, subfamily GH30_8, PDB code 1NOF, rmsd 2.12 Å, in salmon[8]). **c-d,** Identification of conserved clan GH-A residues in the CapGH173 model. In panel (**c**), CapGH173 model is in orange and the GH5 endo-β-1,4-mannanases StMan (PDB code 3WSU) and SACTE_2347 (PDB code 4FK9) are in cyan and green, respectively. In panel (**d**), CapGH173 model is also in orange and the GH30 CpXyn30A and XynA are in blue and salmon, respectively. The residues E305 and E207 correspond to the nucleophile and acid/base, respectively (inferred by structural superposition). Structural alignment was performed using the "super" command in Pymol (The PyMOL Molecular Graphics System, Schrödinger, LLC, New York).

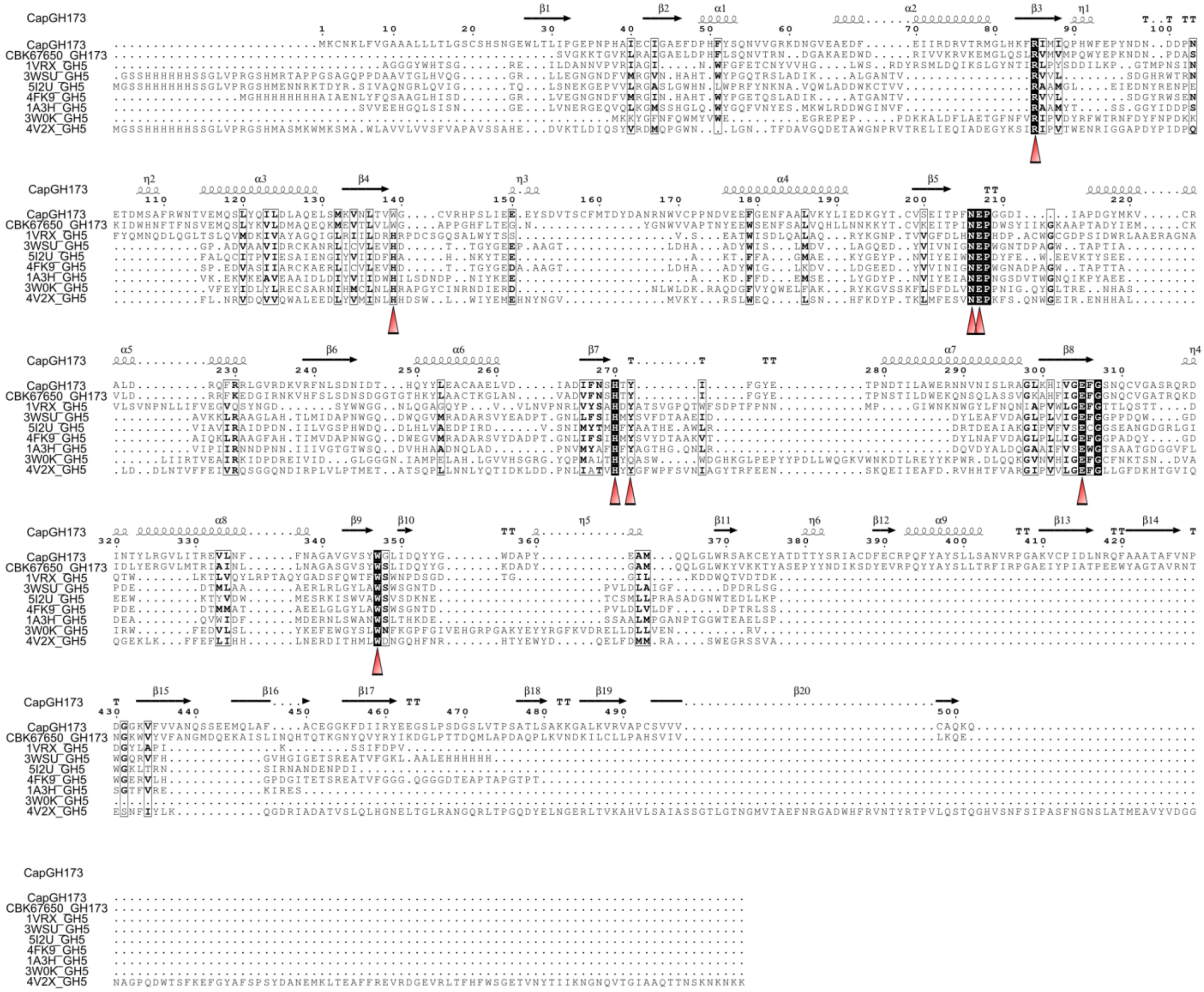

Supplementary Fig. 10. Multiple sequence alignment between GH173 members (CapGH173 and CBK67650 (BXY_26070)) and the GH5 members, described in Supplementary Table 3. Red triangles indicate the conserved residues from Clan GH-A and the inferred catalytic residues. ClustalΩ from MPI Bioinformatics[9] was used to generate the multiple sequence alignments, with manual adjustment based on conserved residues according to structural comparisons. ESPript 3.0[10] was used to generate the alignment image.

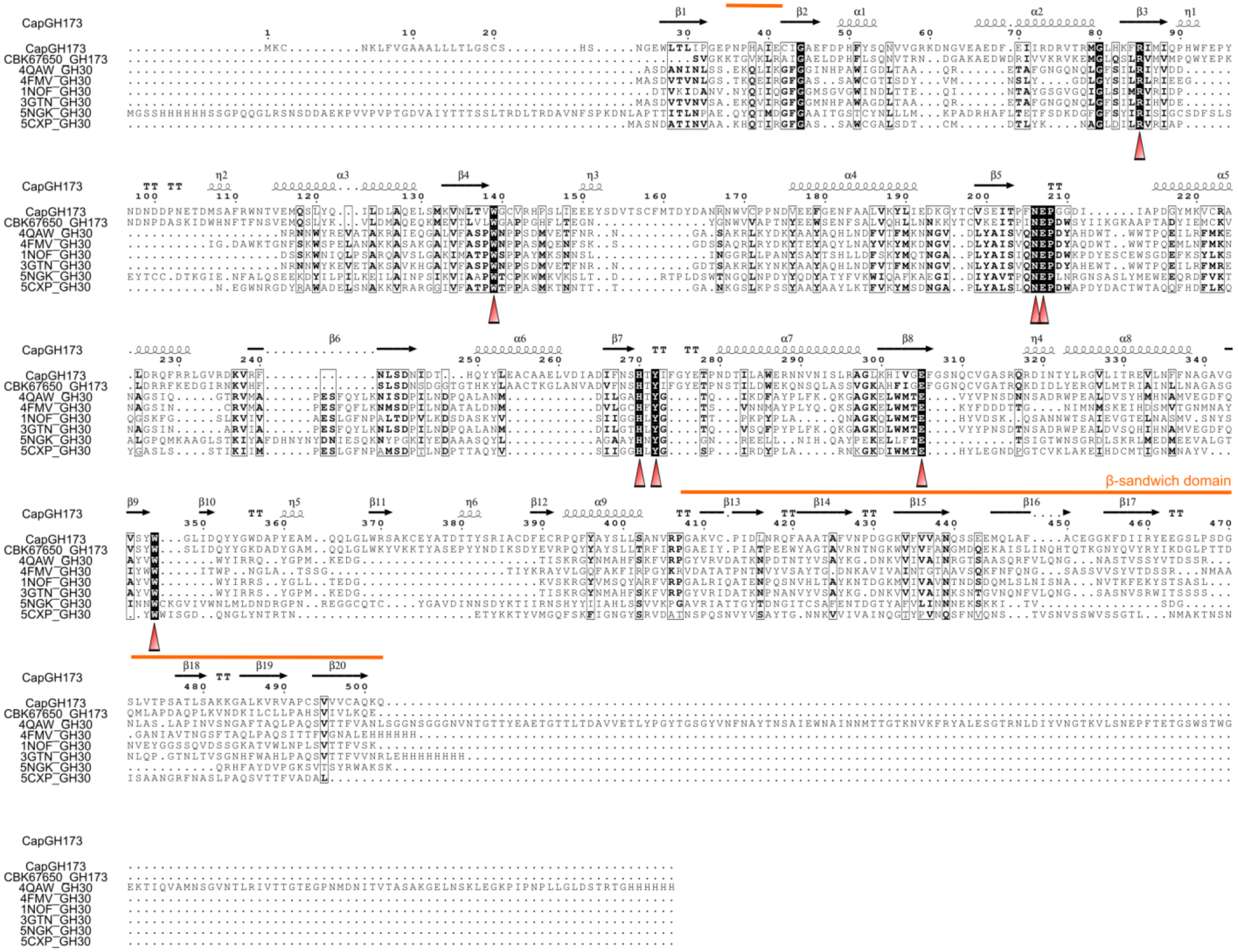

Supplementary Fig. 11. Multiple sequence alignment between GH173 members (CapGH173 and CBK67650 (BXY_26070)) and the GH30 members, described in Supplementary Table 3. Red triangles indicate the conserved residues from Clan GH-A and the inferred catalytic residues. The orange line represents the β-sandwich domain found in GH30 members. ClustalΩ from MPI Bioinformatics[9] was used to generate the multiple sequence alignments, with manual adjustment based on conserved residues according to structural comparisons. ESPript 3.0[10] was used to generate the alignment image.

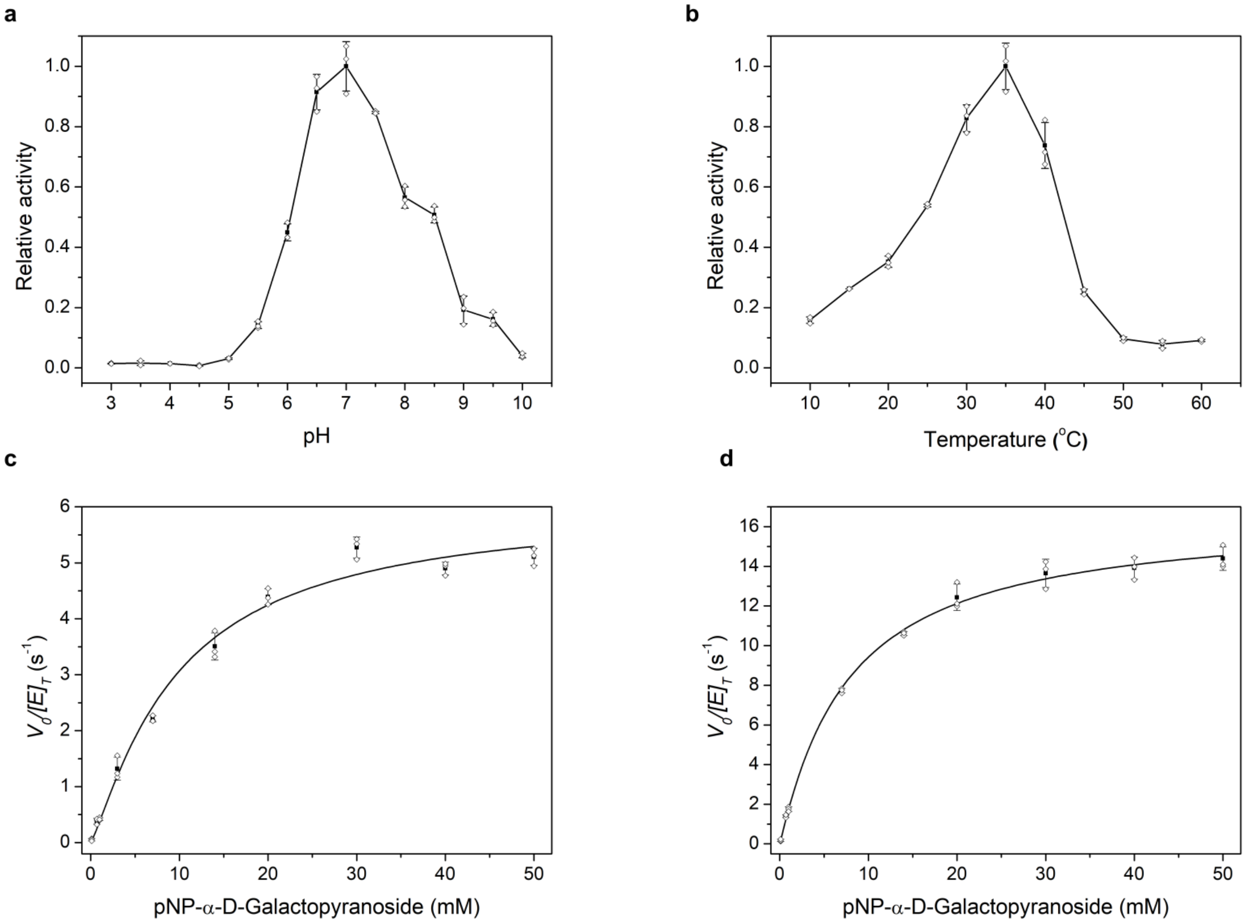

Supplementary Fig. 12. Biochemical and kinetic characterization of CapGH97. Effects of pH (**a**) and temperature (**b**) on the catalytic activity. Kinetic curves of pNP-α-D-Galactopyranoside without $CaCl_2$ (**c**) and with the addition of 5 mM $CaCl_2$ (**d**) assessed at 35 °C and pH 7.0. The kinetic parameters are presented in Table 1. Results are expressed as mean ± SD from three independent experiments (n=3). Data points are shown as empty symbols. Source data are provided as a Source Data file.

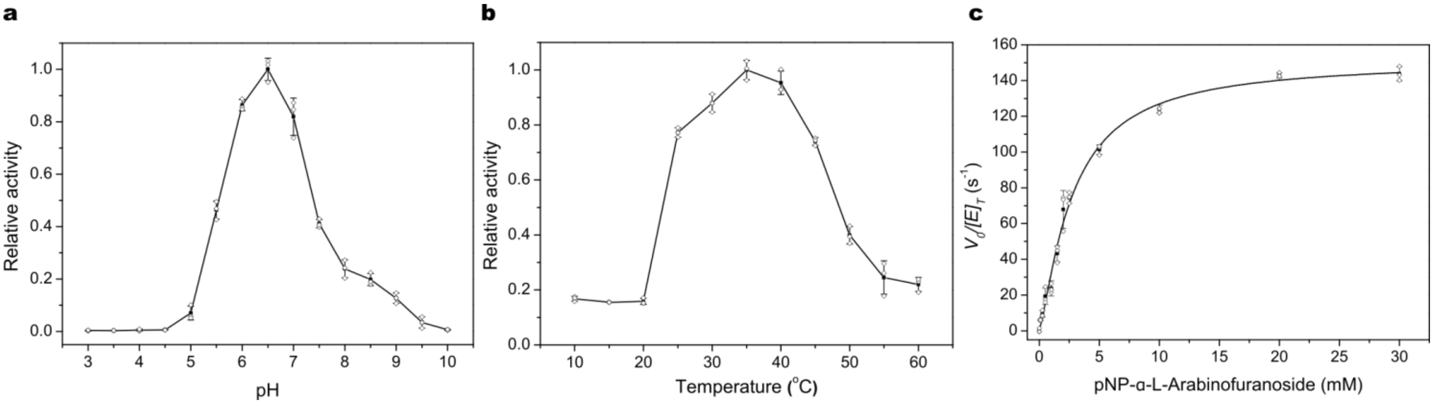

Supplementary Fig. 13. Biochemical characterization of CapGH43_12. Effects of temperature (**a**) and pH (**b**) on the catalytic activity. **c,** Saturation curve using pNP-α-L-Arabinofuranoside as substrate (35 °C and pH 6.5). The kinetic parameters are presented in Table 1. Results are expressed as mean ± SD from three independent experiments (n=3). Data points are shown as empty symbols. Source data are provided as a Source Data file.

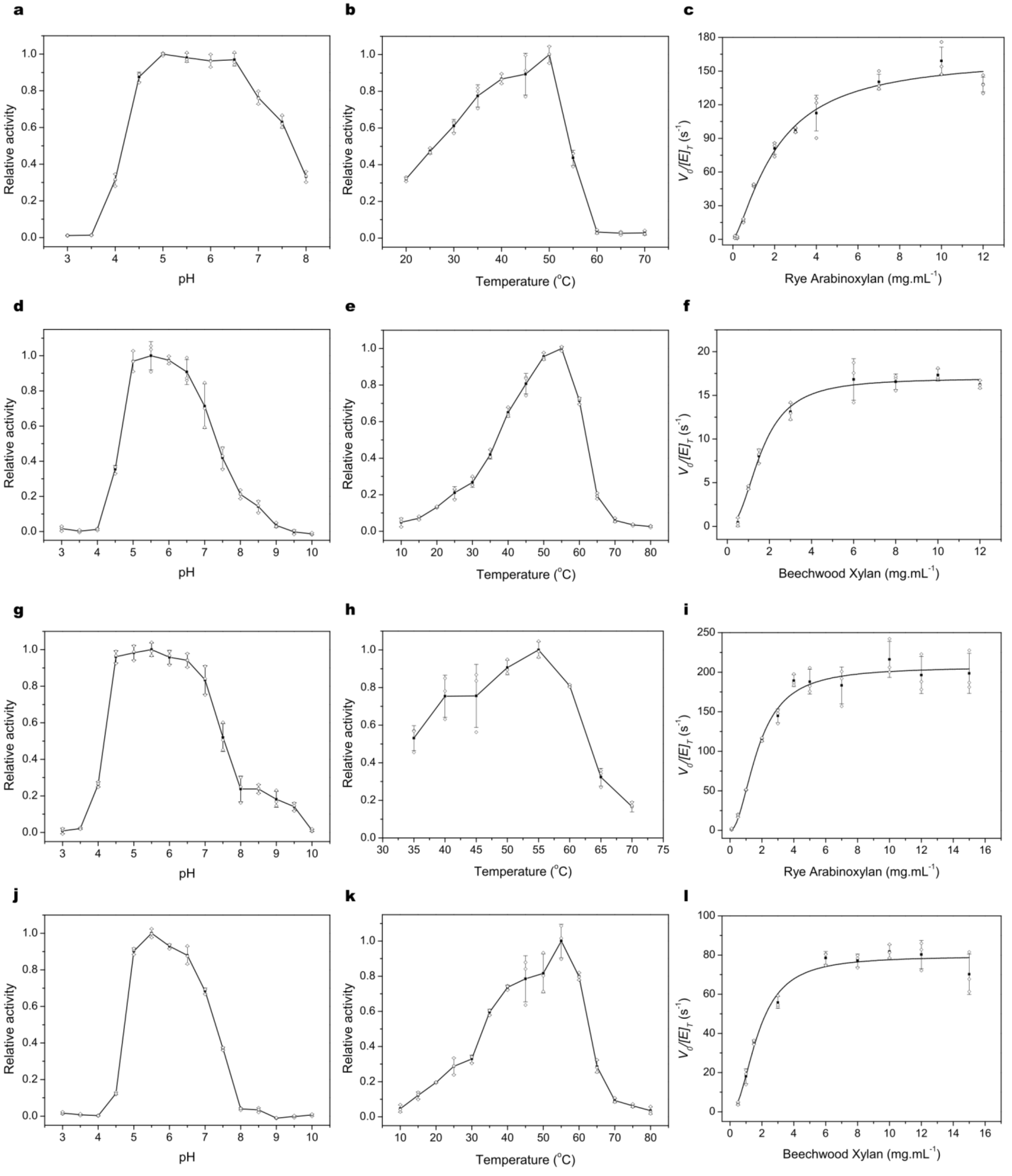

Supplementary Fig. 14. Biochemical and kinetic characterization of CapGH10. Effect of pH and temperature on the catalytic activity and saturation curves. **a-c,** CapGH10 full-length enzyme assayed on rye arabinoxylan at pH 5.0 and 50 °C. **d-f,** CapGH10 full-length enzyme assayed on beechwood xylan at pH 5.5 and 55 °C. **g-i,** CapGH10$_T$ (truncated GH10 catalytic domain) assayed on rye arabinoxylan at pH 5.5 and 55 °C. **j-l,** CapGH10$_T$ assayed on beechwood xylan at pH 5.5 and 55 °C. The kinetic parameters are presented in Table 1. Results are expressed as mean ± SD from three independent experiments (n=3). Data points are shown as empty symbols. Source data are provided as a Source Data file.

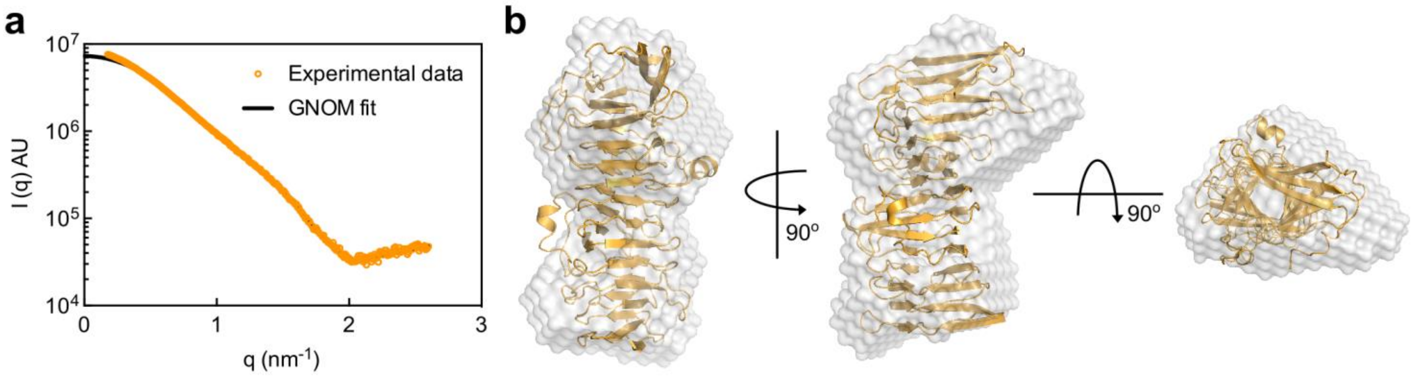

Supplementary Fig. 15. SAXS data of the CapCBM89 domain. a, Experimental SAXS curve (open circles) and theoretical scattering profile (black line) computed from the P(r) function, obtained with GNOM program[11]. (I (q) AU is represented in log scale). **b,** Crystal structure of the CapCBM89 structure fitted into the molecular envelope calculated from SAXS data. Source data are provided as a Source Data file.

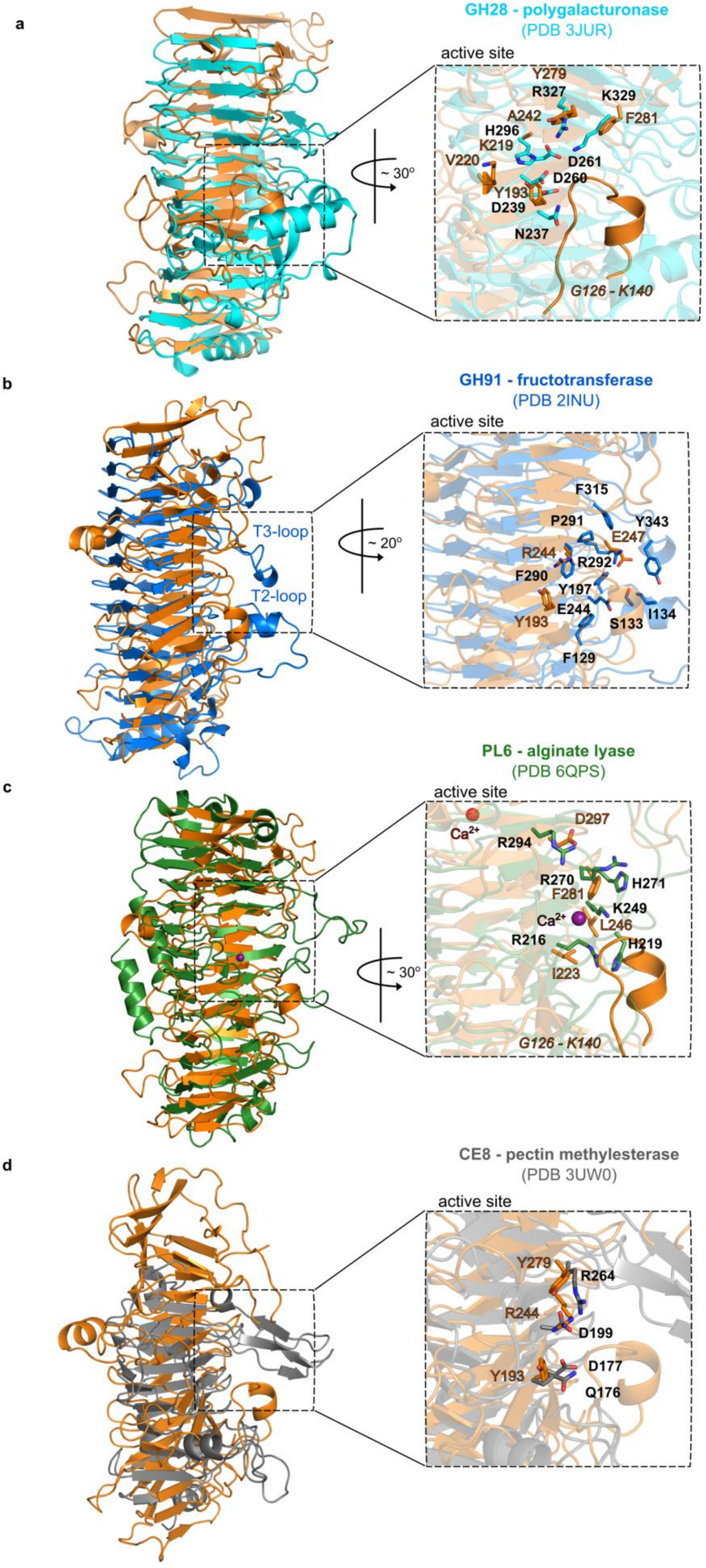

Supplementary Fig. 16. Structural comparisons of CapCBM89 with β-helix Carbohydrate-Active Enzymes database (CAZy) families. a, *Thermotoga maritima* GH28 exo-polygalacturonase (PDB code 3JUR) (cyan[12]). **b**, *Bacillus* sp. GH91 fructotransferase (PDB code 2INU) (blue[13]). **c**, *Bacteroides cellulosilyticus* PL6 alginate lyase (PDB code 6QPS) (green[14]). The Ca^{2+} ions are represented as red (CapCBM89) and purple (alginate lyase) spheres. **d**, *Yersinia enterocolitica* CE8 pectin methylesterase (PDB code 3UW0) (grey[15]). Structures used for comparisons with CapCBM89 were selected according to higher Q-scores shown in Supplementary Table 7. Structural alignments were performed with PDBeFold[16] and/or with Pymol (The PyMOL Molecular Graphics System, Schrödinger, LLC, New York) and manually adjusted to optimize the alignment of cavities.

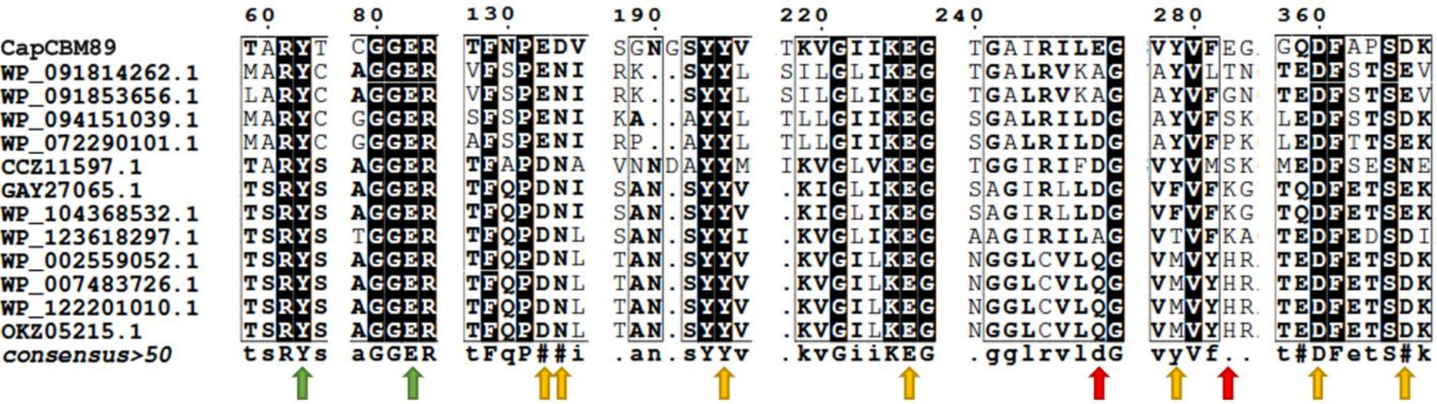

Supplementary Fig. 17. Sequence alignment of CapCBM89 homologues. The protein sequences were obtained by BLASTP search conducted with the isolated CapCBM89 domain. Only sequences containing a possibly GH10 member at the C-terminus were employed for alignment and are specified as their GenBank accession code. Identical residues are highlighted by black background. Red arrows indicate residue mutations that hindered protein expression. Green arrows indicate mutations that altered protein migration pattern in affinity gel electrophoresis (AGE) assays, while yellow arrows indicate mutations that did not affect protein migration in AGE assays. For clarity purposes, only relevant sequence fragments were displayed.

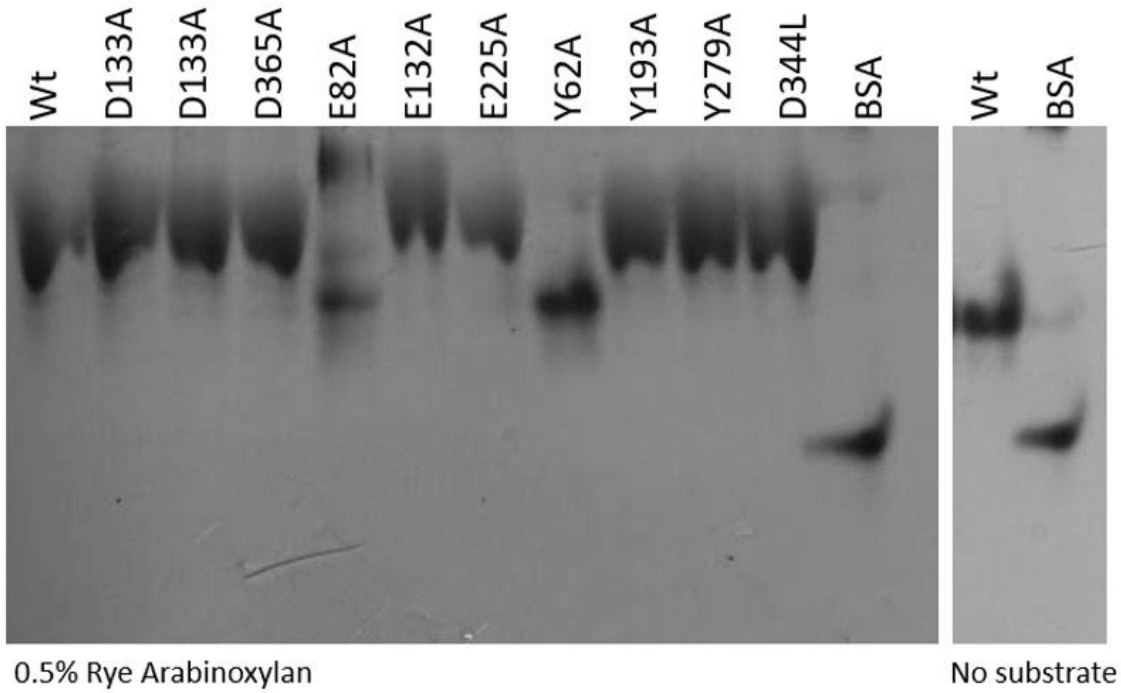

Supplementary Fig. 18. Affinity gel electrophoresis (AGE) of the wild-type CapCBM89 domain (WT) and point mutants. AGE assays were performed with the isolated CapCBM89 domain using bovine serum albumin (BSA) as control. AGE experiments were independently performed three times for CapCBM89 WT (n=3) and twice for mutants (n=2) using arabinoxylan as substrate, with similar results. As the same migration pattern was observed for rye arabinoxylan and beechwood xylan, only results for the rye arabinoxylan are displayed in the figure. By the same reason, only CapCBM89 WT is shown in the control gel, as either WT or mutants migrate equally in AGE without substrates. Source data are provided as a Source Data file.

Supplementary Table 1: Annotation of hypothetical genes evaluated in this study.

GeneID	Annotation	Identity (%)	Expressed in soluble form	Activity	HHpred best hit	HHpred e-value*
**PBMDCECB_19409**	hypothetical protein	14	+	n.d.	Pectinesterase A	3.90E-27
**PBMDCECB_19412**	hypothetical protein	13	+++	n.d.	alginate lyase	1.70E-12
**PBMDCECB_19416**	hypothetical protein	14	+++	n.d.	alginate lyase	1.1E-19
**PBMDCECB_19417**	hypothetical protein	21	n.d.	-	Dextranase	1.3E-49
**PBMDCECB_19418**	hypothetical protein	16	+++	n.d.	Alginate lyase	1.6E-60
**PBMDCECB_19419**	hypothetical protein	15	++	n.d.	LC-Est1C (E.C.3.1.1.1)	9.6e-16
**PBMDCECB_09522**	hypothetical protein	17	+	n.d.	Glycoside hydrolase	1.2E-39
**PBMDCECB_44807**	hypothetical protein	12	+	β-galactosidase	Xylanase B	5.0E-35

*e-value: Expected number of false positives per database search with a score at least as good as the score of the sequence match, calculated by HHpred server https://toolkit.tuebingen.mpg.de/tools/hhpred. n.d. : not detected.

Supplementary Table 2: Isolation source and predicted cellular localization of GH173 family members.

GeneID	Organism	Phylum	Isolation source	SignalP*	BUSCA**
**PBMDCECB_44807**	***Bacteroidales bacterium* MAG42**	**Bacteroidetes**	**Capybara gut microbiome**	**Yes**	**C:extracellular space**
WP_019149822.1	*Alistipes senegalensis*	Bacteroidetes	feces	Yes	C:extracellular space
WP_056620438.1	*Paenibacillus* sp.	Firmicutes	soil	Yes	C:extracellular space
PGCNHMPG_00931	*Bacteroidales bacterium* UBA2817	Bacteroidetes	rumen	Yes	C:cytoplasm
ADELKDKB_00142	*Mageeibacillus* sp. UBA1257	Firmicutes	Switch grass fiber attached community in cow rumen	Yes	C:plasma membrane
PHGEIAKJ_00962	*Prevotella* sp. UBA2732	Bacteroidetes	Rumen fluid	No	C:cytoplasm
CMLMEOMD_03240	*Dysgonomonas* sp. UBA7710	Bacteroidetes	Anode biofilm	Yes	C:extracellular space
DPODELBJ_00546	*Bacteroidales bacterium* UBA1072	Bacteroidetes	feces	Yes	C:extracellular space
IAMDFLOD_01903	*Prevotellaceae bacterium* UBA6382	Bacteroidetes	feces	Yes	C:extracellular space
JMEOHLDH_03232	*Dysgonomonas* sp. UBA7630	Bacteroidetes	metal	Yes	C:cytoplasm
MKLBANBO_00069	*Bacteroidales bacterium* UBA6871	Bacteroidetes	feces	Yes	C:extracellular space
MLCFPEOF_01278	*Proteiniphilum* sp. UBA5492	Bacteroidetes	mud	Yes	C:cytoplasm
OOAECIKD_00412	*Dysgonomonas* sp. UBA7698	Bacteroidetes	Anode biofilm	Yes	C:extracellular space
WP_005845227.1	*Prevotella dentalis*	Bacteroidetes	human oral microbiota	Yes	C:extracellular space
WP_006800034.1	*Dysgonomonas gadei*	Bacteroidetes	human gall bladder	Yes	C:extracellular space
EGN07316.1	*Bacteroides* sp. 1_1_30	Bacteroidetes	human gastrointestinal tract	Yes	C:extracellular space
WP_008998210.1	MULTISPECIES *Bacteroides*	Bacteroidetes	-	Yes	C:extracellular space
WP_010264064.1	*Alistipes timonensis*	Bacteroidetes	feces	Yes	C:extracellular space
WP_019538990.1	*Proteiniphilum acetatigenes*	Bacteroidetes	granule sludge of an upflow anaerobic sludge blanket reactor treating brewery wastewater	No	C:cytoplasm
WP_027953066.1	*Hallella seregens*	Bacteroidetes	oral microbiota	Yes	C:extracellular space
WP_027953076.1	*Hallella seregens*	Bacteroidetes	oral microbiota	Yes	C:extracellular space
WP_032844979.1	MULTISPECIES *Bacteroides*	Bacteroidetes	-	Yes	C:extracellular space
AGB28629.1	*Prevotella dentalis*	Bacteroidetes	human oral microbiota	Yes	C:extracellular space
CBK67650.1	*Bacteroides xylanisolvens*	Bacteroidetes	feces	Yes	C:extracellular space
A0A133Z6P2_9BACT	*Bacteroidales bacterium*	Bacteroidetes	-	Yes	C:extracellular space
A0A139T9T0_9FIRM	*Candidatus Stoquefichus* sp.	Firmicutes	feces	No	C:cytoplasm
A0A196S7T3_BLAHN	*Blastocystis* sp.	Heterokonta	Alga – intestinal parasite	No	C:cytoplasm
A0A1B1FXF0_9BACT	*Flammeovirga* sp.	Bacteroidetes	seawater	Yes	C:extracellular space
A0A1L7I3D6_9FLAO	*Gramella flava*	Bacteroidetes	seawater	Yes	C:extracellular space
A0A268SQ04_9BACL	*Paenibacillus* sp.	Firmicutes	-	Yes	C:cytoplasm

A0A2T7QBW3_9BACT	*Terrimonas* sp.	Bacteroidetes	-	Yes	C:extracellular space
A0A315ZCY6_9BACT	*Sediminitomix flava*	Bacteroidetes	marine sediments	Yes	C:extracellular space
A0A3A9YU17_9ACTN	*Streptomyces hoynatensis*	Actinobacteria	marine sediments	Yes	C:extracellular space
A0A3D9VG82_9ACTN	*Thermasporomyces composti*	Actinobacteria	compost	No	C:cytoplasm
A0A3N2LZ60_9BACT	*Muribaculaceae bacterium*	Bacteroidetes	dominant in the mouse gut microbiota and detected in the intestine of other animals	No	C:cytoplasm
A0A3R6F7M2_9BACT	*Parabacteroides* sp.	Bacteroidetes	feces	Yes	C:extracellular space
A0A4Q6XZE0_9SPHI	*Sphingobacterium* sp.	Bacteroidetes	-	Yes	C:extracellular space
I9S9U4_9BACE	*Bacteroides nordii*	Bacteroidetes	human Intestinal origin	Yes	C:extracellular space
L1N1N3_9BACT	*Prevotella saccharolytica*	Bacteroidetes	human oral cavity	Yes	C:extracellular space
R5BJ25_9BACT	*Alistipes* sp.	Bacteroidetes	intestinal microbiota	Yes	C:cytoplasm
W0IZ67_9BACT	*Opitutaceae bacterium* TAV5	Verrucomicrobia	hindgut of the wood-feeding termite Reticulitermes flavipes	Yes	C:extracellular space
W7Y0J7_9BACT	*Saccharicrinis fermentans*	Bacteroidetes	marine	No	C:cytoplasm

*SignalP - https://services.healthtech.dtu.dk/service.php?SignalP-5.0

**BUSCA - http://busca.biocomp.unibo.it/

Supplementary Table 3: Structural comparison of structurally characterized GH5 and GH30 members, and CapGH173 modelled structure.

Gene ID	Family_Subfamily	Activity	PDB code	Rmsd (Å)*	Identity (%) Coverage (%)
AEY82463.1	GH30_8	glucuronoarabinoxylan-specific endo-β-1,4-xylanase	4QAW	2.06	NS
EGD48159.1	GH30_8	endo-β-1,4-xylanase	4FMV	2.08	NS
AAA75477.1	GH5_1	endo-β-1,4-glucanase	1VRX	2.09	NS
AAB53151.1	GH30_8	glucuronoarabinoxylan-specific endo-β-1,4-xylanase	1NOF	2.12	21% (24%)
CAA97612.1	GH30_8	glucuronoarabinoxylan-specific endo-β-1,4-xylanase	3GTN	2.17	NS
AAO78418.1	GH30_3	endo-β-1,6-glucanase	5NGK	2.75	26% (11%)
AAK76864.1	GH30_8	endo-β-1,4-xylanase	5CXP	3.23	NS
BAK26781.1	GH5_8	endo-β-1,4-mannanase	3WSU	3.27	NS
AND74761.1	GH5_2	endo-β-1,4-glucanase	5I2U	3.55	NS
AEN10237.1	GH5_8	endo-β-1,4-mannanase	4FK9	4.41	26% (20%)
AAC19169.1	GH5_2	endo-β-1,4-glucanase	1A3H	4.54	NS
AGA35556.1	GH5_36	endo-β-1,4-mannanase	3W0K	5.49	NS
BAB04322.1	GH5_4	endo-β-1,4-glucanase	4V2X	5.88	NS

* Structural alignment was performed using the "super" command in Pymol.

NS: No significant similarity found using Blast search with e-value < 1e-5.

Supplementary Table 4: Isolation source and predicted cellular localization of the identified proteins containing a CBM89 domain.

GeneID	Organism	Phylum	Isolation source	SignalP*	BUSCA**
**PBMDCECB_09513**	***Bacteroidaceae bacterium* MAG57**	**Bacteroidetes**	**Capybara gut**	**Yes**	**C:extracellular space**
HCZ20826.1	*Porphyromonadaceae bacterium*	Bacteroidetes	human gut	Yes	C:extracellular space
CDA22303.1	*Bacteroides* sp. CAG:144	Bacteroidetes	Distal human gut microbiota	Yes	C:extracellular space
WP_091814262.1	*Prevotella* sp. BP1-148	Bacteroidetes	rumen microbiome	Yes	C:extracellular space
CCZ11597.1	*Prevotella* sp. CAG:1092	Bacteroidetes	Distal human gut microbiota	Yes	C:cytoplasm
WP_091853656.1	*Prevotella* sp. BP1-145	Bacteroidetes	rumen microbiome	Yes	C:extracellular space
GAY27065.1	*Prevotella* sp. MGM1	Bacteroidetes	mouse gut microbiota	Yes	C:extracellular space
WP_104368532.1	*Prevotella* sp. MGM1	Bacteroidetes	mouse gut microbiota	Yes	C:cytoplasm
WP_094151039.1	*Prevotellaceae bacterium* MN60	Bacteroidetes	rumen microbiome	Yes	C:extracellular space
PWL60328.1	*Bacteroidales bacterium*	Bacteroidetes	human gut	Yes	C:cytoplasm
WP_072290101.1	*Prevotellaceae bacterium* HUN156	Bacteroidetes	rumen microbiome	Yes	C:extracellular space
WP_135042584.1	*Barnesiella* sp. WM24	Bacteroidetes	human gut	Yes	C:cytoplasm
WP_123618297.1	*Muribaculaceae bacterium* Isolate-110 (HZI)	Bacteroidetes	mouse gut microbiota	Yes	C:cytoplasm
WP_002559052.1	*Bacteroides*	Bacteroidetes		Yes	C:extracellular space
WP_007483726.1	*Bacteroides nordii*	Bacteroidetes	human gut	Yes	C:extracellular space
WP_122201010.1	*Bacteroides nordii*	Bacteroidetes	human gut	Yes	C:extracellular space
OKZ05215.1	*Bacteroides* sp. 41_26	Bacteroidetes	human gut	Yes	C:extracellular space
BIMNJMPD_01612	*Bacteroidales bacterium* UBA3663	Bacteroidetes	sheep gut	Yes	C:extracellular space
LOCNPFOL_01009	*Porphyromonadaceae bacterium* UBA7479	Bacteroidetes	Guinea Pig	Yes	C:cytoplasm
KNGJINAD_01644	*Porphyromonadaceae bacterium* UBA7484	Bacteroidetes	Guinea Pig	Yes	C:cytoplasm
NHOEEEMP_00034	*Prevotellaceae bacterium* UBA1710	Bacteroidetes	elephant feces	Yes	C:cytoplasm
EPNIHMFE_00760	*Prevotellaceae bacterium* UBA1716	Bacteroidetes	elephant feces	Yes	C:cytoplasm
NGMNMDAK_01623	*Prevotellaceae bacterium* UBA1726	Bacteroidetes	elephant feces	Yes	C:extracellular space
DEFKBOBF_00387	*Prevotellaceae bacterium* UBA2718	Bacteroidetes	Rumen fluid	Yes	C:extracellular space
CKHBHCDJ_00495	*Prevotellaceae bacterium* UBA2752	Bacteroidetes	Rumen fluid	Yes	C:extracellular space
DOAKLKHJ_01015	*Prevotellaceae bacterium* UBA2852	Bacteroidetes	Rumen	Yes	C:extracellular space
LJCKNPDI_00845	*Prevotellaceae bacterium* UBA2876	Bacteroidetes	Rumen	No	C:cytoplasm
BIPIBPOJ_00907	*Prevotellaceae bacterium* UBA3627	Bacteroidetes	sheep gut	Yes	C:extracellular space
LDFJJJLA_01083	*Prevotellaceae bacterium* UBA3839	Bacteroidetes	sheep gut	No	C:cytoplasm
OELNBKJA_00323	*Prevotellaceae bacterium* UBA4256	Bacteroidetes	sheep gut	No	C:cytoplasm

OGGLENBF_00595	*Prevotellaceae bacterium* UBA4261	Bacteroidetes	sheep gut	Yes	C:extracellular space
CDGGANKO_02361	*Prevotellaceae bacterium* UBA4361	Bacteroidetes	sheep gut	Yes	C:extracellular space
ALFMGCEP_01992	*Prevotellaceae bacterium* UBA4371	Bacteroidetes	sheep gut	Yes	C:extracellular space
FLOJMOOP_00684	*Prevotella* sp. UBA2937	Bacteroidetes	Rumen	Yes	C:extracellular space
MDLLAMAG_01190	*Prevotella* sp. UBA3256	Bacteroidetes	rat gut	Yes	C:extracellular space
LHHCJMON_01197	*Prevotella* sp. UBA3313	Bacteroidetes	rat gut	Yes	C:extracellular space
OBEAGFAE_00968	*Prevotella* sp. UBA3675	Bacteroidetes	sheep gut	Yes	C:extracellular space
FIDEOMML_01975	*Prevotella* sp. UBA3846	Bacteroidetes	sheep gut	Yes	C:extracellular space
JLDLJPLF_01299	*Prevotella* sp. UBA7051	Bacteroidetes	rat gut	Yes	C:extracellular space
OIMLIDDL_00592	*Prevotella* sp. UBA7071	Bacteroidetes	rat gut	Yes	C:extracellular space
PHAJJDNO_01443	*Prevotella* sp. UBA7115	Bacteroidetes	rat gut	Yes	C:extracellular space
MAHBGBIJ_00134	*Prevotella* sp. UBA7140	Bacteroidetes	rat gut	Yes	C:extracellular space
HALFCFOA_01590	*Prevotella* sp. UBA7197	Bacteroidetes	rat gut	Yes	C:extracellular space

*SignalP - https://services.healthtech.dtu.dk/service.php?SignalP-5.0

**BUSCA - http://busca.biocomp.unibo.it/

Supplementary Table 5: Substrates tested against the novel Carbohydrate-Active Enzymes (CAZymes) members. Substrates highlighted in red were used for further assays of the isolated CapGH10 N-terminal domain (CBM89).

Complex substrates	Product code	Company
Arabinan (Sugar Beet)	P-ARAB	Megazyme
Arabinogalactan (Larch Wood)	P-ARGAL	Megazyme
Arabinoxylan (Wheat Flour; Low Viscosity ~ 8 cSt)	P-WAXYL	Megazyme
Avicel® PH-101	11363	Sigma
Carboxymethyl(CM)-Cellulose 4M	P-CMC4M	Megazyme
Chitin	GLU410	ElicityL
Chitosan	448877	Sigma
CM-Curdlan	P-CMCUR	Megazyme
CM-Pachyman	P-CMPAC	Megazyme
Debranched Arabinan	P-DBAR	Megazyme
Galactan (Potato)	P-GALPOT	Megazyme
Galactomannan (Carob; Low Viscosity)	P-GALML	Megazyme
Galactomannan (Guar; Medium Viscosity)	P-GGMMV	Megazyme
Gellan gum	PS113	Dextra
Glucomannan (Konjac; Low Viscosity)	P-GLCML	Megazyme
Glucurono-Arabinoxylan from wheat bran	XYL101	Elicityl
Glucurono-XyloMannan from Tremella fuciformis	HGL200	Elicityl
Laminarin from Laminaria digitata	L9634	Sigma
Lichenan (Icelandic Moss)	P-LICHN	Megazyme
Mannan (Ivory Nut)	P-MANIV	Megazyme
Pectic Galactan (Potato)	P-PGAPT	Megazyme
Pectin from citrus peel	P9135	Sigma
Polygalacturonic Acid	P-PGACT	Megazyme
Pullulan	P-PULLN	Megazyme
Rhamnogalacturonan I (Potato)	P-RHAM1	Megazyme
Scleroglucan	YS09784	Carbosynth
Sodium alginate - low viscosity	YS31736	Carbosynth
Starch from potatoes	S5651	Sigma
Sulfated Arabinogalactan (from Codium f.)	GAL101	Elicityl
Wellan	PS121	Dextra
Xanthan gum from Xanthomonas campestris	G1253	Sigma
Xylan from beechwood	X4252	Sigma
Xyloglucan (Tamarind SEED)	P-XYGLN	Megazyme
β-Glucan (Oat; Medium Viscosity 11CST)	P-BGBL	Megazyme

PNP substrates	Product code	Company
4-Nitrophenyl α-D-galactopyranoside	N0877	Sigma
4-Nitrophenyl α-D-glucopyranoside	N1377	Sigma
4-Nitrophenyl α-D-mannopyranoside	N2127	Sigma
4-Nitrophenyl α-D-xylopyranoside	N1895	Sigma
4-Nitrophenyl α-L-arabinofuranoside	N3641	Sigma
4-Nitrophenyl α-L-arabinopyranoside	N3512	Sigma
4-Nitrophenyl α-L-fucopyranoside	N3628	Sigma
4-Nitrophenyl α-L-ramnopyranoside	N7763	Sigma
4-Nitrophenyl β-D-cellobioside	N5759	Sigma
4-Nitrophenyl β-D-fucopyranoside	N3378	Sigma

4-Nitrophenyl β-D-galactopyranoside	N1252	Sigma
4-Nitrophenyl β-D-glucopyranoside	N7006	Sigma
4-Nitrophenyl β-D-mannopyranoside	N1268	Sigma
4-Nitrophenyl β-D-xylopyranoside	N2132	Sigma
1-Naphthyl butyrate	N8000	Sigma
1-Naphthyl acetate	N8505	Sigma
4-Nitrophenyl acetate	N8130	Sigma
4-Nitrophenyl trans-ferulate	COMH93D5FC7C	Sigma
4-Nitrophenyl formate	6554	Sigma
4-Nitrophenyl myristate	70124	Sigma
4-Nitrophenyl valerate	N4377	Sigma
4-Nitrophenyl palmitate	N2752	Sigma
4-Nitrophenyl decanoate	N0252	Sigma

**Oligosaccharides**	**Product code**	**Company**
Xylotriose	O-XTR	Megazyme
Xylotetrose	O-XTE	Megazyme
Xylopentaose	O-XPE	Megazyme
Xylohexaose	O-XHE	Megazyme
2(2)-(4-O-Methyl-α-D-Glucuronyl)-xylobiose - O-UX	O-UX	Megazyme
2(3)-(4-O-Methyl-α-D-Glucuronyl)-xylotriose - O-UXX	O-UXX	Megazyme
2(2)-(4-O-Methyl-α-D-Glucuronyl)-xylotriose - O-XUX	O-XUX	Megazyme

**Complex hemicelluosic substrates**	**Product origin**
Fucosylated xyloglucan and Glucurono-Xylan (from star fruit)	*donated
Fucosylated xyloglucan and Glucurono-Xylan (from Plum)	*donated
Fucosylated xyloglucan and Xylan (from jambo)	*donated
Fucosylated xyloglucan and Xylan (from soursop)	*donated
Glucurono-Xylan (from guavira)	*donated
Destarched Corn bran	**donated

**Other activity tested**	**Product code**	**Company**
Xylan beechwood (Bromophenol blue assay)	X4252	Sigma
Pectin from Citrus peel (Bromophenol blue assay)	P9135	Sigma
Destarched Corn bran (Bromophenol blue assay)	**donated	
Rhamnogalacturonan I (potato) (Bromophenol blue assay)	P-RHAM1	Megazyme
Glucurono-Arabinoxylan from wheat bran (Bromophenol blue assay)	XYL101	Elicityl
Heparin sodium salt	YH09354	Carbosynth

*Kindly provided by Prof. Lucimara M. C. Cordeiro from Federal University of Parana.

**Kindly provided by Prof. Anne S. Meyer from Technical University of Denmark.

Supplementary Table 6: Data collection and refinement statistics of CapCBM89.

	Native	SeMet derivative
**Data collection**		
Wavelength (Å)	1.033	0.978
Resolution range (Å)	46.38-1.85 (1.92-1.85)	50-2.10 (2.15-2.10)
Space group	$P\ 2_1\ 2_1\ 2_1$	$P\ 2_1\ 2_1\ 2_1$
Unit cell a, b, c (Å)	39.98, 144.51, 169.49	41.80, 143.79, 166.27
Unique reflections	85,143 (8,415)	111,899 (7,713)
Multiplicity	8.8 (9.0)	13.42 (11.26)
Completeness (%)	99.9 (99.9)	98.9 (92.7)
Mean I/sigma(I)	10.37 (0.82)	9.96 (1.59)
R-meas	0.137 (1.919)	0.218 (1.460)
$CC_{1/2}$	0.998 (0.483)	0.997 (0.683)
**Refinement**		
R-work	0.187 (0.345)	
R-free	0.213 (0.338)	
Number of non-hydrogen atoms	6,979	
macromolecules	6,431	
ligands	2	
water	546	
Protein residues	842	
RMS (bonds)	0.008	
RMS (angles)	1.30	
Ramachandran favored (%)	97.0	
Ramachandran outliers (%)	0	
Clash score	2.28	
Average B-factor	47.4	
macromolecules	47.7	
ligands	29	
solvent	44.4	

Statistics for the highest-resolution shell are shown in parentheses.

Supplementary Table 7: Structural alignment of CapCBM89 domain using PDBeFold Server with other β-helix Carbohyrate-Acitive Enzymes (CAZymes) families.

Family	Activity	PDB code	Q-score	Z-score	RMSD	Nalgn	SSM Seq (%)
**GH91**							
	fructotransferase	2INU	0.152	7.878	3.184	233	8.16
	difructose anhydride III hydrolase	5ZKS	0.131	6.833	3.304	232	10.78
**GH28**							
	exopolygalacturonase	3JUR	0.1499	5.034	3.303	249	10.44
	endopolygalacturonase	1CZF	0.1494	5.278	3.164	211	8.53
	polygalacturonase	1BHE	0.1424	5.266	3.349	225	9.33
	endo-xylogalacturonan hydrolase	4C2L	0.1422	4.454	3.545	231	7.79
	endopolygalacturonase	1K5C	0.1308	2.998	3.699	215	7.44
	endopolygalacturonase	1NHC	0.1302	0.544	4.563	247	8.09
	endopolygalacturonase	1HG8	0.13	3.866	3.403	209	9.57
	polygalacturonase	1IA5	0.1268	0.884	4.459	241	9.96
	rhamnogalacturonase	1RMG	0.124	2.985	3.06	212	8.02
	polygalacturonase	2IQ7	0.1231	4.009	3.831	215	7.44
	galacturonidase	5OLP	0.1206	4.997	3.294	220	10
	exopolygalacturonase	2UVE	0.1197	5.374	3.442	257	10.12
	n.d.	4MXN	0.1169	5.811	3.296	156	13.46
**GH49**							
	dextranase	1OGM	0.07796	5.596	3.741	219	5.93
	isopullulanase	1WMR	0.07461	1.804	3.918	216	6.02
	dextranase	6NZS	0.06918	3.087	4.201	225	6.22
**PL1**							
	pectate lyase	2QX3	0.125	5.787	3.272	195	8.72
	pectate lyase	3ZSC	0.1247	5.341	3.132	190	5.79
	pectate lyase	3VMV	0.1238	6.159	3.104	187	9.62
	pectate lyase	2QX3	0.1234	5.836	3.155	190	8.42
	n.d.	1PXZ	0.1162	6.53	3.129	188	4.79
	pectate lyase	1JRG	0.1149	7.122	3.064	187	9.09

	pectate lyase	1BN8	0.1126	5.909	3.016	195	6.67
	pectate lyase	1AIR	0.1103	6.202	3.299	190	8.95
	pectin lyase	1QCX	0.1102	6.275	3.082	185	6.48
	pectin lyase	1IDK	0.1093	5.366	3.009	182	9.89
	pectate lyase	5GT5	0.1068	5.044	3.356	212	8.96
	n.d.	6FI2	0.1051	6.207	3.22	189	8.99
	pectate lyase	4HWV	0.0894	7.821	3.164	199	8.54
	pectate lyase	1VBL	0.0825	4.682	3.704	191	4.71
**PL3**							
	pectate lyase	3T9G	0.1452	3.1	2.664	146	8.9
	pectate lyase	1EE6	0.1266	4.963	3.248	151	3.97
	pectate lyase	3B4N	0.0863	3.941	3.347	160	6.25
	pectate lyase	4U49	0.0824	2.335	3.362	156	9.61
**PL6**							
	alginate lyase	6QPS	0.1723	6.633	3.239	265	7.17
	alginate lyase	5Z9T	0.1331	7.228	3.189	244	9.83
	chondroitinase	1DBG	0.1206	7.878	3.134	226	7.08
	alginate lyase	5GKD	0.0835	6.38	3.488	245	6.53
**PL9**							
	pectate lyase	1RU4	0.1134	5.712	3.168	201	5.97
	rhamnogalacturonan lyase	5OLQ	0.1082	4.842	4.182	239	5.86
**PL31**							
	alginate lyase	6KFN	0.1544	8.122	3.156	202	10.4
**CE8**							
	pectin methylesterase	3UW0	0.1173	5.464	3.335	188	6.38
	pectin methylesterase	2NTQ	0.1166	5.909	3.311	193	6.73
	pectin methylesterase	1QJV	0.1153	5.579	5.579	195	7.18
	pectin methylesterase	5C1C	0.1343	5.964	3.321	194	6.18
	pectin methylesterase	1XG8	0.1326	6.439	3.153	193	6.73
	pectin methylesterase	4PMH	0.1035	7.559	2.946	177	7.34
	pectin methylesterase	1GQ8	0.0983	4.656	3.55	178	10.11
	n.d.	3GRH	0.0826	5.762	3.345	176	9.66

n.d. - not determined

Supplementary Table 8: Number of reads generated by each sample from 16S rRNA gene targeting sequencing (16S), Metagenomics (MG) and Metatranscriptomcics (MT) analyses.

Sample	Omics	#Raw reads	#QC Reads	#QC Reads R1 Only	#QC Reads R2 Only	# Reads removed	# Reads QC merged*	% reads mapped to chloroplasts
16S-Cecum_2-I	16S	47,848	43,123	2,353	2,105	267	28,087	0.03
16S-Cecum_2-II	16S	65,723	61,078	2,522	1,699	424	42,931	0.06
16S-Cecum_2-III	16S	53,557	48,888	2,642	1,618	409	32,433	0.04
16S-Recto_2-I	16S	90,161	82,991	5,083	746	1,341	63,006	0.06
16S-Recto_2-II	16S	99,819	91,369	6,348	730	1,372	67,843	0.06
16S-Recto_2-III	16S	78,542	69,718	6,684	759	1,381	47,522	0.02
16S-Cecum_3-I	16S	103,587	95,147	6,359	654	1,427	73,764	0.14
16S-Cecum_3-II	16S	111,108	102,293	6,798	656	1,361	80,321	0.16
16S-Cecum_3-III	16S	93,676	85,253	6,683	391	1,349	66,611	0.17
16S-Recto_3-I	16S	102,211	90,363	9,651	558	1,639	65,006	0.18
16S-Recto_3-II	16S	113,035	99,931	1,042	591	2,093	72,481	0.18
16S-Recto_3-III	16S	72,901	63,332	8,131	289	1,149	46,287	0.2
16S-Cecum_4-I	16S	89,383	82,467	4,715	917	1,284	61,881	0.06
16S-Cecum_4-II	16S	91,702	84,352	4,723	980	1,647	63,076	0.08
16S-Cecum_4-III	16S	93,738	85,279	6,215	799	1,445	63,872	0.07
16S-Recto_4-I	16S	103,836	92,944	8,426	782	1,684	66,778	0.05
16S-Recto_4-II	16S	88,087	79,375	6,796	574	1,342	58,332	0.04
16S-Recto_4-III	16S	77,590	68,870	6,997	531	1,192	48,958	0.04
Cecum_2_L001	MG	16,053,242	14,927,239	847,953	202,286	75,764	-	0.03
Cecum_2_L002	MG	15,417,083	14,244,944	912,845	181,803	77,491	-	0.03
Cecum_3_L001	MG	16,002,300	14,842,683	867,878	213,157	78,582	-	0.06
Cecum_3_L002	MG	15,266,318	14,074,977	921,775	189,921	79,645	-	0.06
Cecum_4_L001	MG	17,731,418	16,244,891	1,185,884	205,319	95,324	-	0.02
Cecum_4_L002	MG	17,014,212	15,450,579	1,286,779	179,406	97,448	-	0.02
Recto_2_L001	MG	15,863,549	14,701,371	882,912	201,025	78,241	-	0.02
Recto_2_L002	MG	15,231,192	14,053,448	916,169	183,235	7,834	-	0.02
Recto_3_L001	MG	12,145,213	11,548,314	383,947	172,651	40,301	-	0.1
Recto_3_L002	MG	11,684,960	11,091,083	396,796	157,517	39,564	-	0.1
Recto_4_L001	MG	17,010,213	15,981,289	736,022	223,977	68,925	-	0.02
Recto_4_L002	MG	16,289,092	15,263,438	756,556	201,485	67,613	-	0
Cecum_2_L001	MT	26,426,626	23,307,311	1,747,585	630,267	741,463	-	0
Cecum_2_L002	MT	27,179,325	23,842,682	1,900,827	653,389	782,427	-	0
Cecum_3_L001	MT	22,835,041	20,173,224	1,430,662	657,637	573,518	-	0
Cecum_3_L002	MT	23,497,019	20,672,106	1,532,488	685,473	606,952	-	0

Cecum_4_L001	MT	21,352,930	18,984,884	1,378,657	474,823	514,566	-	0
Cecum_4_L002	MT	21,851,007	19,328,451	1,489,788	491,985	540,783	-	0
Recto_2_L001	MT	22,501,589	19,483,042	1,764,248	649,591	604,708	-	0
Recto_2_L002	MT	23,186,062	19,979,906	1,889,135	67,451	642,511	-	0
Recto_3_L001	MT	24,402,376	21,727,516	1,399,893	592,323	682,644	-	0
Recto_3_L002	MT	25,093,469	22,231,815	1,526,303	616,138	719,213	-	0
Recto_4_L001	MT	25,535,199	22,877,975	1,471,999	566,444	618,781	-	0
Recto_4_L002	MT	26,240,441	23,400,599	1,598,569	588,771	652,502	-	0

*For 16S data only

Supplementary Table 9. Summary of sequencing and assembly of reads derived from capybara gut microbiome.

Summary	Capybara co-assembly
No. of contigs (>= 1000 bp)	99,893
Total length (bp)	282,515,049
Largest contig (bp)	252,739
GC (%)	44
N50	3,451
L50	18,207
No. of predicted protein coding genes	257,668
No. of CAZymes protein coding genes	7,377
No. of tRNA	1,968
No. of tmRNA	70
No. of rRNA	57
No. of raw reads MG	185,708,792
No. of QC reads MG	172,424,256
Raw data in Gbases	36
No. of raw reads MT	290,101,084
No. of QC reads MT	256,009,511
Raw data in Gbases	58

Supplementary Table 10: Description of Sequence Read Archive (SRA) accession numbers of each sample.

Omics	Accession	Sample description	Link
MG	SRR11852049	Capybara gut microbiome Cecum MG rep 2	https://www.ncbi.nlm.nih.gov/sra/SRR11852049
MG	SRR11852057	Capybara gut microbiome Cecum MG rep 2	https://www.ncbi.nlm.nih.gov/sra/SRR11852057
MG	SRR11852048	Capybara gut microbiome Cecum MG rep 3	https://www.ncbi.nlm.nih.gov/sra/SRR11852048
MG	SRR11852056	Capybara gut microbiome Cecum MG rep 3	https://www.ncbi.nlm.nih.gov/sra/SRR11852056
MG	SRR11852047	Capybara gut microbiome Cecum MG rep 4	https://www.ncbi.nlm.nih.gov/sra/SRR11852047
MG	SRR11852053	Capybara gut microbiome Cecum MG rep 4	https://www.ncbi.nlm.nih.gov/sra/SRR11852053
MG	SRR11852046	Capybara gut microbiome Rectum MG rep 2	https://www.ncbi.nlm.nih.gov/sra/SRR11852046
MG	SRR11852052	Capybara gut microbiome Rectum MG rep 2	https://www.ncbi.nlm.nih.gov/sra/SRR11852052
MG	SRR11852051	Capybara gut microbiome Rectum MG rep 3	https://www.ncbi.nlm.nih.gov/sra/SRR11852051
MG	SRR11852055	Capybara gut microbiome Rectum MG rep 3	https://www.ncbi.nlm.nih.gov/sra/SRR11852055
MG	SRR11852050	Capybara gut microbiome Rectum MG rep 4	https://www.ncbi.nlm.nih.gov/sra/SRR11852050
MG	SRR11852054	Capybara gut microbiome Rectum MG rep 4	https://www.ncbi.nlm.nih.gov/sra/SRR11852054
MT	SRR11852100	Capybara gut microbiome Cecum MT rep 2	https://www.ncbi.nlm.nih.gov/sra/SRR11852100
MT	SRR11852104	Capybara gut microbiome Cecum MT rep 2	https://www.ncbi.nlm.nih.gov/sra/SRR11852104
MT	SRR11852099	Capybara gut microbiome Cecum MT rep 3	https://www.ncbi.nlm.nih.gov/sra/SRR11852099
MT	SRR11852103	Capybara gut microbiome Cecum MT rep 3	https://www.ncbi.nlm.nih.gov/sra/SRR11852103
MT	SRR11852102	Capybara gut microbiome Cecum MT rep 4	https://www.ncbi.nlm.nih.gov/sra/SRR11852102
MT	SRR11852108	Capybara gut microbiome Cecum MT rep 4	https://www.ncbi.nlm.nih.gov/sra/SRR11852108
MT	SRR11852101	Capybara gut microbiome Rectum MT rep 2	https://www.ncbi.nlm.nih.gov/sra/SRR11852101
MT	SRR11852107	Capybara gut microbiome Rectum MT rep 2	https://www.ncbi.nlm.nih.gov/sra/SRR11852107
MT	SRR11852098	Capybara gut microbiome Rectum MT rep 3	https://www.ncbi.nlm.nih.gov/sra/SRR11852098
MT	SRR11852106	Capybara gut microbiome Rectum MT rep 3	https://www.ncbi.nlm.nih.gov/sra/SRR11852106
MT	SRR11852097	Capybara gut microbiome Rectum MT rep 4	https://www.ncbi.nlm.nih.gov/sra/SRR11852097
MT	SRR11852105	Capybara gut microbiome Rectum MT rep 4	https://www.ncbi.nlm.nih.gov/sra/SRR11852105
16S	SRR11852076	Capybara gut microbiome Cecum 16S rep 2 I	https://www.ncbi.nlm.nih.gov/sra/SRR11852076
16S	SRR11852085	Capybara gut microbiome Cecum 16S rep 2 II	https://www.ncbi.nlm.nih.gov/sra/SRR11852085
16S	SRR11852086	Capybara gut microbiome Cecum 16S rep 2 III	https://www.ncbi.nlm.nih.gov/sra/SRR11852086
16S	SRR11852070	Capybara gut microbiome Cecum 16S rep 3 I	https://www.ncbi.nlm.nih.gov/sra/SRR11852070
16S	SRR11852071	Capybara gut microbiome Cecum 16S rep 3 II	https://www.ncbi.nlm.nih.gov/sra/SRR11852071
16S	SRR11852072	Capybara gut microbiome Cecum 16S rep 3 III	https://www.ncbi.nlm.nih.gov/sra/SRR11852072
16S	SRR11852073	Capybara gut microbiome Cecum 16S rep 4 I	https://www.ncbi.nlm.nih.gov/sra/SRR11852073

16S	SRR11852074	Capybara gut microbiome Cecum 16S rep 4 II	https://www.ncbi.nlm.nih.gov/sra/SRR11852074
16S	SRR11852075	Capybara gut microbiome Cecum 16S rep 4 III	https://www.ncbi.nlm.nih.gov/sra/SRR11852075
16S	SRR11852069	Capybara gut microbiome Rectum 16S rep 2 I	https://www.ncbi.nlm.nih.gov/sra/SRR11852069
16S	SRR11852083	Capybara gut microbiome Rectum 16S rep 2 II	https://www.ncbi.nlm.nih.gov/sra/SRR11852083
16S	SRR11852084	Capybara gut microbiome Rectum 16S rep 2 III	https://www.ncbi.nlm.nih.gov/sra/SRR11852084
16S	SRR11852077	Capybara gut microbiome Rectum 16S rep 3 I	https://www.ncbi.nlm.nih.gov/sra/SRR11852077
16S	SRR11852078	Capybara gut microbiome Rectum 16S rep 3 II	https://www.ncbi.nlm.nih.gov/sra/SRR11852078
16S	SRR11852079	Capybara gut microbiome Rectum 16S rep 3 III	https://www.ncbi.nlm.nih.gov/sra/SRR11852079
16S	SRR11852080	Capybara gut microbiome Rectum 16S rep 4 I	https://www.ncbi.nlm.nih.gov/sra/SRR11852080
16S	SRR11852081	Capybara gut microbiome Rectum 16S rep 4 II	https://www.ncbi.nlm.nih.gov/sra/SRR11852081
16S	SRR11852082	Capybara gut microbiome Rectum 16S rep 4 III	https://www.ncbi.nlm.nih.gov/sra/SRR11852082

Supplementary Table 11: Metagenome-assembled genomes (MAGs) NCBI accession numbers.

ID	Biosample ID	GenomeID	Link
**MAG1**	SAMN15049459	JABUSA000000000	https://www.ncbi.nlm.nih.gov/search/all/?term=JABUSA000000000
**MAG2**	SAMN15049460	JABUSB000000000	https://www.ncbi.nlm.nih.gov/search/all/?term=JABUSB000000000
**MAG3**	SAMN15049461	JABUSC000000000	https://www.ncbi.nlm.nih.gov/search/all/?term=JABUSC000000000
**MAG4**	SAMN15049462	JABUSD000000000	https://www.ncbi.nlm.nih.gov/search/all/?term=JABUSD000000000
**MAG5**	SAMN15049463	JABUSE000000000	https://www.ncbi.nlm.nih.gov/search/all/?term=JABUSE000000000
**MAG6**	SAMN15049464	JABUSF000000000	https://www.ncbi.nlm.nih.gov/search/all/?term=JABUSF000000000
**MAG7**	SAMN15049465	JABUSG000000000	https://www.ncbi.nlm.nih.gov/search/all/?term=JABUSG000000000
**MAG8**	SAMN15049466	JABUSH000000000	https://www.ncbi.nlm.nih.gov/search/all/?term=JABUSH000000000
**MAG9**	SAMN15049467	JABUSI000000000	https://www.ncbi.nlm.nih.gov/search/all/?term=JABUSI000000000
**MAG10**	SAMN15049468	JABUSJ000000000	https://www.ncbi.nlm.nih.gov/search/all/?term=JABUSJ000000000
**MAG11**	SAMN15049469	JABUSK000000000	https://www.ncbi.nlm.nih.gov/search/all/?term=JABUSK000000000
**MAG12**	SAMN15049470	JABUSL000000000	https://www.ncbi.nlm.nih.gov/search/all/?term=JABUSL000000000
**MAG13**	SAMN15049471	JABUSM000000000	https://www.ncbi.nlm.nih.gov/search/all/?term=JABUSM000000000
**MAG14**	SAMN15049472	JABUSN000000000	https://www.ncbi.nlm.nih.gov/search/all/?term=JABUSN000000000
**MAG15**	SAMN15049473	JABUSO000000000	https://www.ncbi.nlm.nih.gov/search/all/?term=JABUSO000000000
**MAG16**	SAMN15049474	JABUSP000000000	https://www.ncbi.nlm.nih.gov/search/all/?term=JABUSP000000000
**MAG17**	SAMN15049475	JABUSQ000000000	https://www.ncbi.nlm.nih.gov/search/all/?term=JABUSQ000000000
**MAG18**	SAMN15049476	JABUSR000000000	https://www.ncbi.nlm.nih.gov/search/all/?term=JABUSR000000000
**MAG19**	SAMN15049477	JABUSS000000000	https://www.ncbi.nlm.nih.gov/search/all/?term=JABUSS000000000
**MAG20**	SAMN15049478	JABUST000000000	https://www.ncbi.nlm.nih.gov/search/all/?term=JABUST000000000
**MAG21**	SAMN15049479	JABUSU000000000	https://www.ncbi.nlm.nih.gov/search/all/?term=JABUSU000000000
**MAG22**	SAMN15049480	JABUSV000000000	https://www.ncbi.nlm.nih.gov/search/all/?term=JABUSV000000000
**MAG23**	SAMN15049481	JABUSW000000000	https://www.ncbi.nlm.nih.gov/search/all/?term=JABUSW000000000
**MAG24**	SAMN15049482	JABUSX000000000	https://www.ncbi.nlm.nih.gov/search/all/?term=JABUSX000000000
**MAG25**	SAMN15049483	JABUSY000000000	https://www.ncbi.nlm.nih.gov/search/all/?term=JABUSY000000000
**MAG26**	SAMN15049484	JABUSZ000000000	https://www.ncbi.nlm.nih.gov/search/all/?term=JABUSZ000000000
**MAG27**	SAMN15049485	JABUTA000000000	https://www.ncbi.nlm.nih.gov/search/all/?term=JABUTA000000000
**MAG28**	SAMN15049486	JABUTB000000000	https://www.ncbi.nlm.nih.gov/search/all/?term=JABUTB000000000
**MAG29**	SAMN15049487	JABUTC000000000	https://www.ncbi.nlm.nih.gov/search/all/?term=JABUTC000000000
**MAG30**	SAMN15049488	JABUTD000000000	https://www.ncbi.nlm.nih.gov/search/all/?term=JABUTD000000000
**MAG31**	SAMN15049489	JABUTE000000000	https://www.ncbi.nlm.nih.gov/search/all/?term=JABUTE000000000
**MAG32**	SAMN15049490	JABUTF000000000	https://www.ncbi.nlm.nih.gov/search/all/?term=JABUTF000000000
**MAG33**	SAMN15049491	JABUTG000000000	https://www.ncbi.nlm.nih.gov/search/all/?term=JABUTG000000000

**MAG34**	SAMN15049492	JABUTH000000000	https://www.ncbi.nlm.nih.gov/search/all/?term=JABUTH000000000
**MAG35**	SAMN15049493	JABUTI000000000	https://www.ncbi.nlm.nih.gov/search/all/?term=JABUTI000000000
**MAG36**	SAMN15049494	JABUTJ000000000	https://www.ncbi.nlm.nih.gov/search/all/?term=JABUTJ000000000
**MAG37**	SAMN15049495	JABUTK000000000	https://www.ncbi.nlm.nih.gov/search/all/?term=JABUTK000000000
**MAG38**	SAMN15049496	JABUTL000000000	https://www.ncbi.nlm.nih.gov/search/all/?term=JABUTL000000000
**MAG39**	SAMN15049497	JABUTM000000000	https://www.ncbi.nlm.nih.gov/search/all/?term=JABUTM000000000
**MAG40**	SAMN15049498	JABUTN000000000	https://www.ncbi.nlm.nih.gov/search/all/?term=JABUTN000000000
**MAG41**	SAMN15049499	JABUTO000000000	https://www.ncbi.nlm.nih.gov/search/all/?term=JABUTO000000000
**MAG42**	SAMN15049500	JABUTP000000000	https://www.ncbi.nlm.nih.gov/search/all/?term=JABUTP000000000
**MAG43**	SAMN15049501	JABUTQ000000000	https://www.ncbi.nlm.nih.gov/search/all/?term=JABUTQ000000000
**MAG44**	SAMN15049502	JABUTR000000000	https://www.ncbi.nlm.nih.gov/search/all/?term=JABUTR000000000
**MAG45**	SAMN15049503	JABUTS000000000	https://www.ncbi.nlm.nih.gov/search/all/?term=JABUTS000000000
**MAG46**	SAMN15049504	JABUTT000000000	https://www.ncbi.nlm.nih.gov/search/all/?term=JABUTT000000000
**MAG47**	SAMN15049505	JABUTU000000000	https://www.ncbi.nlm.nih.gov/search/all/?term=JABUTU000000000
**MAG48**	SAMN15049506	JABUTV000000000	https://www.ncbi.nlm.nih.gov/search/all/?term=JABUTV000000000
**MAG49**	SAMN15049507	JABUTW000000000	https://www.ncbi.nlm.nih.gov/search/all/?term=JABUTW000000000
**MAG50**	SAMN15049508	JABUTX000000000	https://www.ncbi.nlm.nih.gov/search/all/?term=JABUTX000000000
**MAG51**	SAMN15049509	JABUTY000000000	https://www.ncbi.nlm.nih.gov/search/all/?term=JABUTY000000000
**MAG52**	SAMN15049510	JABUTZ000000000	https://www.ncbi.nlm.nih.gov/search/all/?term=JABUTZ000000000
**MAG53**	SAMN15049511	JABUUA000000000	https://www.ncbi.nlm.nih.gov/search/all/?term=JABUUA000000000
**MAG54**	SAMN15049512	JABUUB000000000	https://www.ncbi.nlm.nih.gov/search/all/?term=JABUUB000000000
**MAG55**	SAMN15049513	JABUUC000000000	https://www.ncbi.nlm.nih.gov/search/all/?term=JABUUC000000000
**MAG56**	SAMN15049514	JABUUD000000000	https://www.ncbi.nlm.nih.gov/search/all/?term=JABUUD000000000
**MAG57**	SAMN15049515	JABUUE000000000	https://www.ncbi.nlm.nih.gov/search/all/?term=JABUUE000000000
**MAG58**	SAMN15049516	JABUUF000000000	https://www.ncbi.nlm.nih.gov/search/all/?term=JABUUF000000000
**MAG59**	SAMN15049517	JABUUG000000000	https://www.ncbi.nlm.nih.gov/search/all/?term=JABUUG000000000
**MAG60**	SAMN15049518	JABUUH000000000	https://www.ncbi.nlm.nih.gov/search/all/?term=JABUUH000000000
**MAG61**	SAMN15049519	JABUUI000000000	https://www.ncbi.nlm.nih.gov/search/all/?term=JABUUI000000000
**MAG62**	SAMN15049520	JABUUJ000000000	https://www.ncbi.nlm.nih.gov/search/all/?term=JABUUJ000000000
**MAG63**	SAMN15049521	JABUUK000000000	https://www.ncbi.nlm.nih.gov/search/all/?term=JABUUK000000000
**MAG64**	SAMN15049522	JABUUL000000000	https://www.ncbi.nlm.nih.gov/search/all/?term=JABUUL000000000
**MAG65**	SAMN15049523	JABUUM000000000	https://www.ncbi.nlm.nih.gov/search/all/?term=JABUUM000000000
**MAG66**	SAMN15049524	JABUUN000000000	https://www.ncbi.nlm.nih.gov/search/all/?term=JABUUN000000000
**MAG67**	SAMN15049525	JABUUO000000000	https://www.ncbi.nlm.nih.gov/search/all/?term=JABUUO000000000
**MAG68**	SAMN15049526	JABUUP000000000	https://www.ncbi.nlm.nih.gov/search/all/?term=JABUUP000000000
**MAG69**	SAMN15049527	JABUUQ000000000	https://www.ncbi.nlm.nih.gov/search/all/?term=JABUUQ000000000

**MAG70**	SAMN15049528	JABUUR000000000	https://www.ncbi.nlm.nih.gov/search/all/?term=JABUUR000000000
**MAG71**	SAMN15049529	JABUUS000000000	https://www.ncbi.nlm.nih.gov/search/all/?term=JABUUS000000000
**MAG72**	SAMN15049530	JABUUT000000000	https://www.ncbi.nlm.nih.gov/search/all/?term=JABUUT000000000
**MAG73**	SAMN15049531	JABUUU000000000	https://www.ncbi.nlm.nih.gov/search/all/?term=JABUUU000000000
**MAG74**	SAMN15049532	JABUUV000000000	https://www.ncbi.nlm.nih.gov/search/all/?term=JABUUV000000000
**MAG75**	SAMN15049533	JABUUW000000000	https://www.ncbi.nlm.nih.gov/search/all/?term=JABUUW000000000
**MAG76**	SAMN15049534	JABUUX000000000	https://www.ncbi.nlm.nih.gov/search/all/?term=JABUUX000000000
**MAG77**	SAMN15049535	JABUUY000000000	https://www.ncbi.nlm.nih.gov/search/all/?term=JABUUY000000000
**MAG78**	SAMN15049536	JABUUZ000000000	https://www.ncbi.nlm.nih.gov/search/all/?term=JABUUZ000000000
**MAG79**	SAMN15049537	JABUVA000000000	https://www.ncbi.nlm.nih.gov/search/all/?term=JABUVA000000000

Supplementary References

1. Parks, D. H. *et al.* Recovery of nearly 8,000 metagenome-assembled genomes substantially expands the tree of life. *Nat. Microbiol.* **2**, 1533–1542 (2017).
2. Na, S.-I. I. *et al.* UBCG: Up-to-date bacterial core gene set and pipeline for phylogenomic tree reconstruction. *J. Microbiol.* **56**, (2018).
3. Baek, M. *et al.* Accurate prediction of protein structures and interactions using a three-track neural network. *Science* **373**, 871–876 (2021).
4. Laskowski, R. A., Jabłońska, J., Pravda, L., Vařeková, R. S. & Thornton, J. M. PDBsum: structural summaries of PDB entries. *Protein Sci.* **27**, 129–134 (2018).
5. Kumagai, Y. *et al.* The loop structure of Actinomycete glycoside hydrolase family 5 mannanases governs substrate recognition. *FEBS J.* **282**, 4001–4014 (2015).
6. Takasuka, T. E. *et al.* Biochemical properties and atomic resolution structure of a proteolytically processed β-mannanase from cellulolytic *Streptomyces* sp. SirexAA-E. *PLoS One* **9**, e94166 (2014).
7. St John, F. J. *et al.* A novel member of glycoside hydrolase family 30 subfamily 8 with altered substrate specificity. *urn:issn:1399-0047* **70**, 2950–2958 (2014).
8. Larson, S. B., Day, J., De la Rosa, A. P. B., Keen, N. T. & McPherson, A. First crystallographic structure of a xylanase from glycoside hydrolase family 5: implications for catalysis. *Biochemistry* **42**, 8411–8422 (2003).
9. Gabler, F. *et al.* Protein sequence analysis using the MPI bioinformatics toolkit. *Curr. Protoc. Bioinforma.* **72**, e108 (2020).
10. Robert, X. & Gouet, P. Deciphering key features in protein structures with the new ENDscript server. *Nucleic Acids Res.* **42**, W320–W324 (2014).
11. Svergun, D. I. Determination of the regularization parameter in indirect-transform methods using perceptual criteria. *J. Appl. Crystallogr.* **25**, 495–503 (1992).
12. Pijning, T., van Pouderoyen, G., Kluskens, L., van der Oost, J. & Dijkstra, B. W. The crystal structure of a hyperthermoactive exopolygalacturonase from *Thermotoga maritima* reveals a unique tetramer. *FEBS Lett.* **583**, 3665–3670 (2009).
13. Jung, W. S. *et al.* Structural and functional insights into intramolecular fructosyl transfer by inulin fructotransferase. *J. Biol. Chem.* **282**, 8414–8423 (2007).
14. Stender, E. G. P. *et al.* Structural and functional aspects of mannuronic acid–specific PL6 alginate lyase from the human gut microbe *Bacteroides cellulosilyticus. J. Biol. Chem.* **294**, 17915–17930 (2019).
15. Boraston, A. B. & Abbott, D. W. Structure of a pectin methylesterase from *Yersinia enterocolitica. Acta Crystallogr. Sect. F Struct. Biol. Cryst. Commun.* **68**, 129–133 (2012).
16. Krissinel, E. & Henrick, K. Secondary-structure matching (SSM), a new tool for fast protein structure alignment in three dimensions. *Acta Crystallogr. Sect. D Biol. Crystallogr.* **60**, 2256–2268 (2004).
