# Supplementary material for: Gut microbiome of the largest living rodent harbors unprecedented enzymatic systems to degrade plant polysaccharides
Source: Nat Commun. 2022 Feb 2;13:629. doi: 10.1038/s41467-022-28310-y (PMC8810776; doi:10.1038/s41467-022-28310-y)
Supplement: Supplementary file 3 — Description of Additional Supplementary Files [file 41467_2022_28310_MOESM3_ESM.docx]

**Description of Additional Supplementary Files**

**File Name:** Supplementary Data 1

**Description:** Genome information of the metagenome-assembled genomes (MAGs) recovered from Capybara gut microbiota.

**File Name:** Supplementary Data 2

**Description:** Number of carbohydrate-active enzymes (CAZymes) in each metagenome-assembled genome (MAG) recovered from capybara gut microbiome. MAG57 is highlighted in yellow because this genome encompasses the largest inventory of CAZymes among recovered MAGs.

**File Name:** Supplementary Data 3

**Description:** Multi-modular carbohydrate-active enzymes (CAZymes) identified in the capybara gut microbiome.

**File Name:** Supplementary Data 4

**Description:** Supplementary Data 4: Summarized expression of main carbohydrate-active enzymes (CAZymes) from capybara metagenome-assembed genomes (MAGs).

**File Name:** Supplementary Data 5

**Description:** Polysaccharides utilization loci (PULs) and clusters of CAZymes (CCs) identified in the metagenome-assembled genomes (MAGs).

**File Name:** Supplementary Data 6

**Description:** Nuclear magnetic resonance (NMR) based metabolomics of capybara gut samples. Concentration are expressed in μM.

**File Name:** Supplementary Data 7

**Description:** Expression of genes related to dietary components fermentation into short-chain fatty acids (SCFAs) in the recovery metagenomeassembled genomes (MAGs).
