# Supplementary material for: Gut microbiome of the largest living rodent harbors unprecedented enzymatic systems to degrade plant polysaccharides
Source: Nat Commun. 2022 Feb 2;13:629. doi: 10.1038/s41467-022-28310-y (PMC8810776; doi:10.1038/s41467-022-28310-y)
Supplement: Supplementary file 11 — Reporting Summary [file 41467_2022_28310_MOESM11_ESM.pdf]

nature portfolio

Corresponding author(s): Mario T. Murakami and Gabriela F. Persinoti

Last updated by author(s): 2022/01/13

Reporting Summary

Nature Portfolio wishes to improve the reproducibility of the work that we publish. This form provides structure for consistency and transparency in reporting. For further information on Nature Portfolio policies, see our Editorial Policies and the Editorial Policy Checklist.

Statistics

For all statistical analyses, confirm that the following items are present in the figure legend, table legend, main text, or Methods section.

n/a	Confirmed	
[ ]	[x]	The exact sample size (*n*) for each experimental group/condition, given as a discrete number and unit of measurement
[ ]	[x]	A statement on whether measurements were taken from distinct samples or whether the same sample was measured repeatedly
[ ]	[x]	The statistical test(s) used AND whether they are one- or two-sided<br>*Only common tests should be described solely by name; describe more complex techniques in the Methods section.*
[x]	[ ]	A description of all covariates tested
[x]	[ ]	A description of any assumptions or corrections, such as tests of normality and adjustment for multiple comparisons
[ ]	[x]	A full description of the statistical parameters including central tendency (e.g. means) or other basic estimates (e.g. regression coefficient) AND variation (e.g. standard deviation) or associated estimates of uncertainty (e.g. confidence intervals)
[ ]	[x]	For null hypothesis testing, the test statistic (e.g. *F*, *t*, *r*) with confidence intervals, effect sizes, degrees of freedom and *P* value noted<br>*Give P values as exact values whenever suitable.*
[x]	[ ]	For Bayesian analysis, information on the choice of priors and Markov chain Monte Carlo settings
[x]	[ ]	For hierarchical and complex designs, identification of the appropriate level for tests and full reporting of outcomes
[x]	[ ]	Estimates of effect sizes (e.g. Cohen's *d*, Pearson's *r*), indicating how they were calculated

Our web collection on statistics for biologists contains articles on many of the points above.

Software and code

Policy information about availability of computer code

Data collection	High-throughput sequencing of 16S rRNA gene was performed on Illumina Miseq System. Metagenomics (MG) and Metatranscriptomics (MT) high-throughput sequencing was performed on Illumina Hiseq 2500 system and fastq files were generated using the bcl2fastq v2.18.12. Long reads were generated on MinION device using a Flow cell R9 version and fast5 files were converted to fastq files using Guppy basecaller V. 3.2.2+9fr0a78.<br>NMR data were collect using a Varian Inova NMR spectrometer (Agilent).<br>Crystallographic data were collected using Pilatus 2M detector (Dectris) and MXCuBE 2 software at MX2-LNLS and using a DECTRIS EIGER 9M detector (Dectris) and MxCuBE 2 software at PROXIMA-2A-SOLEIL. SAXS data were collected using a Pilatus 300K (Dectris) detector and fit2D software.<br>Spectrophotometric data were collected using the i-Control software (1.10.4.0) (Tecan).
Data analysis	Processing of 16S rRNA gene amplicon was done with a combination of standalone programs, Trimmomatic v.0.36, Usearch v.10, and phyloseq v1.20 R package, R 3.6.3 and RStudio v. 1.3.1093.<br><br>MT reads were also analyzed using SortmeRNA v. 2.0 to remove rRNA reads. Both MG and MT reads were taxonomically classified using Kaiju v. 1.7.4. Reago v.1.1 was used for recovery 16S ribosomal RNA from the MG data.<br><br>MG trimmed reads were de novo co-assembled using IDBA_UD v. 1.1.1. The assembled metagenome was binned using CONCOCT v.0.4.0 and MaxBin 2.0 and dRep v. 2.0.5 was used to dereplicate the multiple assembly and binning combinations to produce an optimal set of MAGs. Completeness and contamination was determined for each MAG using CheckM v1.0.6. Long-reads sequencing (ONT) were used for MAGs scaffolding using SSPACE-long-reads v1.1. MAGs taxonomy was assigned using GTDB-tk tool v.1.4 was used with the release 202 of the GTDB database. Gene prediction and annotation of both the recovered genomes and the co-assembly were performed using Prokka v.1.11. KEGG pathways and Kegg Orthologous (KOs) annotation were performed using KOFAMscan v. 1.3.0 and Functional Ontology Assignments for Metagenomes (FOAM) database (e-value < 1e-5). Annotation for CAZymes were performed using the CAZy annotation pipeline with libraries

from July 2019. Kallisto v. 0.46.1 was used to estimate the coverage/abundance of protein coding genes in cecal and rectal samples, expressed as TPM (Transcripts per Million).

CAZymes phylogenetic analysis was carried out using the catalytic domain of each family aligned with MAFFT v. 7.475 , and using maximum likelihood methods implemented in the RAxML v. 8.2.12.

Metabolic reconstruction analysis was performed using the AMON software v 1.0.0.

Structural models of the CapGH173, GH10 domain from the CapGH10, CapGH97 and CapGH43_12 were obtained using RoseTTAFold, available in the Robetta structure prediction server. Protein topology of CapGH173 was obtained using PDBsum server.

NMR data were processed using Chenomx NMR Suite 7.6 software (Chenomx Inc.). SAXS data were integrated using Fit2D (18). The programs Fit2D (18 beta), GNOM (4.6), DAMMIF (1.1), and SUPCOMB (2.3) were used for SAXS data processing.

Crystallographic data were indexed and scaled using XDS (version Jan 26th 2018 Built 20180808). Single-wavelenght anomalous diffraction data were processed using SHELXC/D/E from CCP4i package 7.0.023. The AutoBuild wizard from the Phenix package (dev-3139) was used for initial model build. Refinements were carried out with phenix.refine (1.8.3) and Refmac (5.8), using COOT (0.8.9) for manual building. Model validations were done using Molprobity (4.5) and PDBRedo server (https://pdb-redo.eu/). Figures containing crystallographic coordinates were generated using Pymol (2.3 or 1.3).

OriginPro (8) was used for enzymatic non-linear curves fitting.

For manuscripts utilizing custom algorithms or software that are central to the research but not yet described in published literature, software must be made available to editors and reviewers. We strongly encourage code deposition in a community repository (e.g. GitHub). See the Nature Portfolio guidelines for submitting code & software for further information.

Data

Policy information about availability of data

All manuscripts must include a data availability statement. This statement should provide the following information, where applicable:

- Accession codes, unique identifiers, or web links for publicly available datasets
- A description of any restrictions on data availability
- For clinical datasets or third party data, please ensure that the statement adheres to our policy

All sequencing data generated in this study can be found under the BioProject ID PRJNA563062 (https://www.ncbi.nlm.nih.gov/bioproject/PRJNA563062). The 16S, metagenomic and metatranscriptomic reads for cecal and rectal samples have been deposited in the Sequence Read Archive (SRA) under the accession numbers SRR11852069-SRR11852086, SRR11852046-SRR11852057 and SRR11852097-SRR11852108, respectively (Supplementary Table 10). The recovered MAGs have been deposited in the GenBank under the accession numbers JABUSA000000000-JABUVA000000000 (Supplementary Table S11). The NMR metabolomics data have been deposited in the Metabolomics Workbench database under accession number ST001945 (http://dx.doi.org/10.21228/M8G11Z). Atomic coordinates and structure factors have been deposited in the Protein Data Bank (PDB) under accession code 7JVI (CapCBM89) (10.2210/pdb7JVI/pdb). Other data generated or analyzed during this study are included in this published article and its Supplementary information files. Source data are provided with this paper.
Data and code used for microbiome analyses are publicly available at https://github.com/gpersinoti/capybara_microbiome.

Field-specific reporting

Please select the one below that is the best fit for your research. If you are not sure, read the appropriate sections before making your selection.

☒ Life sciences ☐ Behavioural & social sciences ☐ Ecological, evolutionary & environmental sciences

For a reference copy of the document with all sections, see nature.com/documents/nr-reporting-summary-flat.pdf

Life sciences study design

All studies must disclose on these points even when the disclosure is negative.

Sample size	No sample size calculation was performed in advance for the exploratory analysis of the microbial community associated with capybara gut microbiome. The number of enzymatic independent experiments was determined based upon previous studies with similar methodologies (doi.org/10.1038/s41589-020-0554-5), following the standard practice in enzymology (n=3).
Data exclusions	No data were excluded from the analyses.
Replication	The authors believe that within the constraints of working with wild animals, the conclusions in the manuscript are supported with sufficient replicates and sample analysis as well as with multiple datasets (16S rRNA gene analysis, metagenomics and metatranscriptomics). All sample sizes are indicated in the respective figure legends. Quantitative enzyme assays consist of three independent experiments (n=3). Affinity gel electrophoresis (AGE) experiments were performed three times (n=3) for the CapCBM89 wild-type (WT), and twice (n=2) for mutants. Omics analyses were conducted with three biological independent samples (n=3) from three different animals.
Randomization	In each experiment, the samples were treated identically. Therefore, randomization was not relevant.

Blinding	In each experiment, the samples were treated identically. Therefore, blinding was not relevant.

Reporting for specific materials, systems and methods

We require information from authors about some types of materials, experimental systems and methods used in many studies. Here, indicate whether each material, system or method listed is relevant to your study. If you are not sure if a list item applies to your research, read the appropriate section before selecting a response.

Materials & experimental systems

n/a	Involved in the study
☒	☐ Antibodies
☒	☐ Eukaryotic cell lines
☒	☐ Palaeontology and archaeology
☐	☒ Animals and other organisms
☒	☐ Human research participants
☒	☐ Clinical data
☒	☐ Dual use research of concern

Methods

n/a	Involved in the study
☒	☐ ChIP-seq
☒	☐ Flow cytometry
☒	☐ MRI-based neuroimaging

Animals and other organisms

Policy information about studies involving animals; ARRIVE guidelines recommended for reporting animal research

Laboratory animals	The study did not involve laboratory animals.
Wild animals	The samples were collected from three young female capybara (Hydrochoerus hydrochaeris) specimens.<br><br>The sample collection for this study was performed during one of the campaigns conducted by Nunes et al., 2020 (10.1590/0103-8478cr2020053), which focused on the reproductive control of capybaras at risk of transmission of Rocky Mountain Spotted Fever (RMSF). Briefly, animals were captured at Tatui/SP (September 2017), and blood samples were collected and the serum was tested for the presence of anti-R. rickettsii antibodies by immunofluorescence assays. The seropositive animals were considered immune for developing rickettsemia, then underwent surgical procedures of tubal ligations in non-gestational females and vasectomy in males, to impair the reproduction of these animals. On the other hand, seronegative animals were euthanized as a strategy to control the proliferation of the bacteria R. rickettsia, a practice that is guaranteed by Brazilian Joint Resolution SMA/SES No. 1, of July 2016. Since we aimed to collect fresh samples directly from the cecum and recto, we only collected samples from the seronegative animals for R. rickettsii, which were euthanized.
Field-collected samples	The study did not involved samples collected from the field.
Ethics oversight	This study was carried out in strict accordance with the Animal Management Rule of the Brazilian Ministry of Environment (Sisbio 59826-1).

Note that full information on the approval of the study protocol must also be provided in the manuscript.
